# Electrically Induced Mixed Valence Increases the Conductivity of Copper Helical Metallopolymers

**DOI:** 10.1002/adma.202100403

**Published:** 2021-05-06

**Authors:** Jake L. Greenfield, Daniele Di Nuzzo, Emrys W. Evans, Satyaprasad P. Senanayak, Sam Schott, Jason T. Deacon, Adele Peugeot, William K. Myers, Henning Sirringhaus, Richard H. Friend, Jonathan R. Nitschke

**Affiliations:** ^1^ Department of Chemistry University of Cambridge Lensfield Road Cambridge CB2 1EW UK; ^2^ Cavendish Laboratory University of Cambridge JJ Thomson Avenue Cambridge CB3 0HE UK; ^3^ Centre for Advanced ESR Department of Chemistry University of Oxford South Parks Road Oxford OX1 3QR UK

**Keywords:** chirality, metallopolymers, mixed‐valency, resistive switching, self‐assembly

## Abstract

Controlling the flow of electrical current at the nanoscale typically requires complex top‐down approaches. Here, a bottom‐up approach is employed to demonstrate resistive switching within molecular wires that consist of double‐helical metallopolymers and are constructed by self‐assembly. When the material is exposed to an electric field, it is determined that ≈25% of the copper atoms oxidize from Cu^I^ to Cu^II^, without rupture of the polymer chain. The ability to sustain such a high level of oxidation is unprecedented in a copper‐based molecule: it is made possible here by the double helix compressing in order to satisfy the new coordination geometry required by Cu^II^. This mixed‐valence structure exhibits a 10^4^‐fold increase in conductivity, which is projected to last on the order of years. The increase in conductivity is explained as being promoted by the creation, upon oxidation, of partly filled $d_{z^{2}}$ orbitals aligned along the mixed‐valence copper array; the long‐lasting nature of the change in conductivity is due to the structural rearrangement of the double‐helix, which poses an energetic barrier to re‐reduction. This work establishes helical metallopolymers as a new platform for controlling currents at the nanoscale.

## Graphical abstract

Modulation in the conductivity of 1D materials is of importance for nanoscale control of electrical currents. Self‐assembled molecular wires containing Cu ions in the +1 oxidation state are shown to increase their conductivity by 4 orders of magnitude upon applying an electrical stimulus. The mechanism of this resistance change is attributed to the system adopting a mixed‐valence Cu^I^/Cu^II^ state.

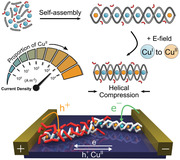

## 1 Introduction

Organic‐based molecular materials offer the structural complexity necessary to build functionality at the nanoscale and from the bottom up.^[^ ^1^, ^2^, ^3^ ^]^ In hybrid metal–organic materials, the oxidation state of metal ions provides a further handle to engineer new properties.^[^ ^4^, ^5^, ^6^, ^7^, ^8^, ^9^, ^10^, ^11^ ^]^ Manipulation of the metal oxidation state in such systems impacts their electrical properties: Switching of bulk resistivity in thin films has indeed been demonstrated via changing the oxidation state of the metal centers in metallopolymers.^[^ ^7^, ^9^, ^10^ ^]^ In these reports, however, the metal ions are not organized into well‐defined structures and are often separated from each other by organic moieties.^[^ ^4^, ^5^, ^6^, ^7^, ^8^, ^9^, ^10^, ^11^ ^]^ This results in limited metal–metal communication, and ultimately in poor nanoscale control of the metal system, due to the inherent disorder in polymer films.

An appealing approach to create ordered structures at the nanoscale is to place the metal centers in direct communication with each other.^[^ ^12^ ^]^ To this end, systems that contain linear chains of metal ions have been reported.^[^ ^2^, ^6^, ^13^, ^14^, ^15^, ^16^, ^17^, ^18^, ^19^, ^20^, ^21^, ^22^, ^23^, ^24^, ^25^ ^]^ Of these, extended metal atom chains (EMACs) are molecules in which the metal ions in the chain are electronically coupled, permitting the development of novel nanoelectronic devices.^[^ ^14^, ^15^, ^16^, ^24^ ^]^ The oxidation state of the metal ions in these materials has a critical impact on the electrical conductivity, with mixed‐valence along the array creating a delocalized electronic system.^[^ ^15^, ^16^, ^22^ ^]^ While these previous works have investigated the impact of native mixed‐valence on the electronic properties, however, external control of the oxidation state remains to be explored, and exploited, in functional devices.

Here, we describe the electrical behavior of a self‐assembled, solution‐processable double‐helical metallopolymer, which consists of a linear array of Cu^I^ ions coordinated to two organic polymeric ligand strands. Methods have been developed to control the regiochemistry, enantiomeric excess, and lengths of such polymers.^[^ ^26^, ^27^ ^]^ Upon exposure to an electric field, we find the polymer to adopt a novel mixed‐valence structure, wherein ≈25% of the copper atoms in the chain oxidize from Cu^I^ to Cu^II^, without rupture of the polymer chain. Instead, the helically wrapped ligand strands compress and wind more tightly about the Cu^II^ centers, adapting to the preferred geometry of the metal ion in this oxidation state and taking up radical‐anion character to balance the increased charge on the metal core. This process results in an increase in conductivity of thin films by four orders of magnitude, and the more conductive state is extrapolated to last for several years at 300 K. We conclude that the increase in conductivity is promoted by efficient charge transport via partly filled $d_{z^{2}}$ orbitals extending along the mixed‐valence copper array, and that the long‐lasting nature of the high‐conductivity state is due to structural re‐organization of the polymer chain, which presents an energetic barrier to Cu^II^ re‐reduction. This linear metallopolymer is thus an EMAC which can be rendered conductive using an external electrical stimulus. Our work introduces copper double‐helical metallopolymers as an easily processable system, based on the earth‐abundant metal copper, for the bottom‐up creation and control of conductive atomic chains.

## 2 Results

**Figure 1:**
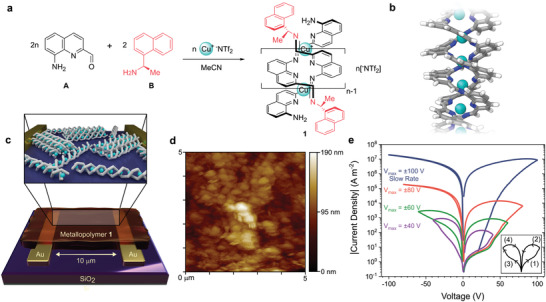
Self‐assembly of metallopolymer and resistive switching. a) Subcomponent self‐assembly of helical metallopolymer **1** from monomer **A**, end‐cap **B**, and Cu^I^. The metallopolymer employed, unless otherwise stated, is a 50mer, with head‐to‐tail regiochemistry and a 52% enrichment of *M* handedness. b) Schematic representation of **1**, depicting a pair of conjugated polymer strands wrapped helically around a linear array of Cu^I^ centres. c) The device architecture employed to study charge transport through a 50 nm thickness of **1**. The inset depicts a schematic representation of **1** dispersed between the gold electrodes. d) AFM height image of a thin film deposited under the same coating conditions as for the devices, highlighting that the film consists of slightly elongated domains of metallopolymer **1** with an average size for the long side of 420 nm (Figure S4, Supporting Information). e) Absolute value of current density versus voltage for a 50mer of metallopolymer **1**, showing the higher currents obtained as the device was scanned to progressively higher maximum voltages (*V*_max_). The arrows and numbers in the inset show how the voltage was modulated during each set of scans: first positive from zero, then back through zero and negative, then back to zero again. For the set where *V*_max_ = 100 V, the scan time between 0 and *V*_max_ was 115 s, for a total of 460 s for the four consecutive scans. For all the other curves it was 15 s, for a total of 60 s for the four scans. The different sets of measurements, indicated as different colours, were performed on fresh, nonpoled devices. The same electrical behavior was observed on glass substrates. The substrates were washed and re‐used several times, showing no sign of damage to the SiO_2_ layer caused by operation.

Copper‐based double‐helical metallopolymer **1** was prepared by self‐assembly of monomer **A** (100 equiv) with a small amount of end‐cap **B** (2 equiv) and CuNTf_2_ (50.5 equiv; Tf = O_2_SCF_3_) in MeCN (**Figure** **1**a,b).^[^ ^26^, ^27^ ^]^ We estimate the spacing between the Cu^I^ centers within **1** to be 0.3 nm based upon the crystal structure of a corresponding oligomer^[^ ^26^ ^]^ and electron micrographs of the polymer in an aggregated state.^[^ ^27^ ^]^ The **B** termini have been shown both to serve as chain‐capping agents to control polymer length, and to dictate the handedness of the helical polymer strands;^[^ ^27^ ^]^ in this study an **A**‐ to ‐**B** ratio was chosen to produce chains of *M* handedness (>52% helical excess, measured using circular dichroism)^[^ ^27^ ^]^ that were an average of 50 repeat units long (measured using dynamic light scattering).^[^ ^27^ ^]^ Such oligomers were found to self‐organize into tightly packed intermolecular aggregates,^[^ ^27^ ^]^ the formation of which is favored by enantiomeric excess.

The co‐existence of twin conjugated organic strands with a linear array of redox‐active metal ions within **1** raise the question of which pathway would electrical charges follow in this polymer. To shed light on this question, we set out to investigate the electrical properties in the solid state. Thin films of **1** were prepared by spin coating, showing domains with an average size of 420 nm (Figure 1d, Figure S4, Supporting Information). Within these domains we observed high aspect ratio rod‐like structures tens of nm long and averaging 10 nm across, oriented preferentially in the plane of the substrate (Figure S5, Supporting Information).^[^ ^27^ ^]^ To investigate the electrical properties of **1** in the plane of the metallopolymer chains, we therefore constructed in‐plane devices as shown in Figure 1c, consisting of two gold electrodes deposited on silicon dioxide, separated by a distance of 10 µm and with electrode widths of 700 µm, onto which we deposited thin (50 nm) films of **1**.

We performed current–voltage scans (Figure 1e) and observed a combination of transient and long‐lasting effects, with the current density depending on the scan speed and on the maximum absolute voltage reached (*V*_max_). Taking each time a fresh device, four current‐voltage scans were performed, as noted in the inset of Figure 1e. Scans (1) and (2) trace the forward and return scans, respectively, with positive voltage bias applied, and scans (3) and (4) represent the forward and return scans under negative voltage bias (Figure 1e, inset). For *V*_max_ values of 40 and 60 V, both positive and negative bias scans showed substantial hysteresis, with the current densities in (2) and (4) significantly higher than in (1) and (3), respectively. For *V*_max_ = 80 V, however, scans (3) and (4) showed negligible hysteresis and higher currents than the corresponding forward scans (1) and (2). When scans were performed at a slower rate (0.6 V s^–1^) reaching *V*_max_ = 100 V, the current density was observed to reach the same values in (2) as in (3) and (4), as well as displaying a linear dependence on voltage above 50 V (Figure S6, Supporting Information). This linear dependence indicates that the device undergoes ohmic injection and transport above 50 V: Under these conditions, a bulk conductivity of 2 S m^–1^ was estimated (Section S4.1, Supporting Information). Despite the high potentials employed, given the distance between the electrodes (10 µm) the electric field applied across the metallopolymer films is moderate (10^7 ^V m^–1^ at 100 V, comparable to the field applied to an OLED under standard operating conditions).

These results are rationalized as follows. The initial effect of the external electric field is to drive ion motion, and the ^−^NTf_2_ counteranions migrate to the positively charged electrode to generate surface‐charge‐dipole (SCD) regions at the electrodes. These regions can facilitate charge injection, as is observed in devices such as light‐emitting electrochemical cells.^[^ ^28^ ^]^ In the slower 100 V scan of Figure 1e, the current density during sweep (1) deviates from the trend observed in the faster scans, indicating that ion migration has had time to take place and facilitate charge injection. The effects of ion migration are transient, causing device properties to return to their initial conditions when the voltage bias is switched off. When the applied voltage is sufficiently high, or at lower scan rates (80 and 100 V curves, Figure 1e), an additional, long‐lasting change in the material is observed, which causes the devices to enter a low resistivity state independent of the scan polarity, removing hysteresis and asymmetry between scans in Figure 1e. This resistive switching effect is inferred to result from a structural change taking place in the bulk of the material, as described below.

**Figure 2:**
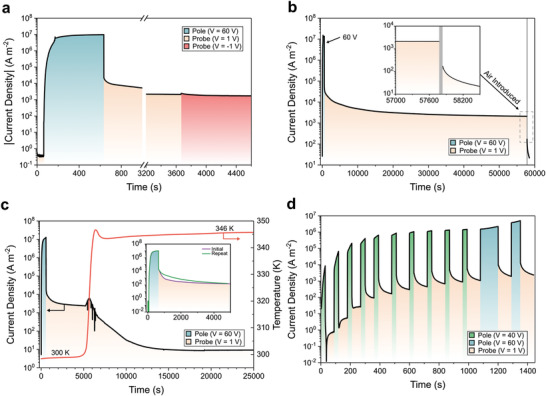
Resistive switching under different operating conditions. Current density as a function of time and voltage bias recorded on devices having the structure shown in Figure 1c, containing metallopolymer **1**. Unless specified, the measurements were performed at constant temperature (300 K) and under vacuum (10^–5^ bar). a) Evolution following the application of subsequent “probe” (1 or −1 V) and “pole” (60 V) voltage steps. The *y*‐axis shows the absolute current density, in order to display the results obtained at different voltage polarities. b) Evolution on a longer time scale, with the device exposed to air after 16 h biased at 1 V. c) Effect of temperature on current density. The inset shows the reversibility of the effect: After heating the device to 346 K, the current density recovered to the same value as was observed for the pristine material. d) Sequential pole and probe cycles showing the different resistance states of the device accessed as a function of total pole duration; the current density value plateaued at ≈900 s when using 40 V pole voltage and increased further following the application of a 60 V pole voltage.

To probe the resistive switching behavior of these devices in more detail, we investigated the current density as a function of time using different applied voltages. The application of 1 V between the two gold contacts to a pristine device resulted in a current density of ≈0.5 A m^–2^ (**Figure** **2**a). Increase of the potential to 60 V caused the current density to increase rapidly, reaching a plateau at ≈10^7^ A m^–2^. Consistent with the results described above (Figure 1e, slow scan, *V*_max_ = ±100 V), this current density corresponds to a conductivity of 1.7 S m^–1^ (Section 4.1, Supporting Information). When the potential across the device was switched to 1 V, the current density settled between 10^3^ and 10^4^ A m^–2^, four orders of magnitude higher than in the pristine state (i.e., the state before the 60 V “pole” step was applied). Inverting the potential to −1 V (after 1 h at +1 V) did not affect the current density, indicating that the conductance of the device is independent of the polarity of the “probe” voltage. This polarity independence implies that the change in current density induced by electric poling is related to a change in the bulk conductivity of the material as opposed to a change in the injection properties. Note that in Figure 2a the devices were poled for 570 s at a constant voltage bias of 60 V. These conditions represent a significantly longer and stronger poling than in the short scans of Figure 1e (60 s for the four consecutive scans between +60 and −60 V): Indeed, full and long‐lasting resistive switching is observed in Figure 1e only when a slow scan is used (460 s in total, reaching ±100 V).

We observed the high‐conductivity state obtained after poling at 60 V to remain with negligible loss for over 16 h (Figure 2b); we fitted the current density decay in the 1 V probe step to a power law *a*/*t^b^* + *c*, where *c* is fixed at 0.5 A m^–2^. Extrapolating this decay shows that under our experimental conditions (1 V, 300 K, in vacuum) the high conductivity state could in principle be retained for years (Figure S8, Supporting Information).^[^ ^29^ ^]^

Exposing the poled device to air brought about a rapid decrease in current density (Figure 2b). Measurements performed on devices that had been exposed to air while in the poled state showed a much slower build‐up of current at 60 V, reaching a plateau at 1000 times lower current density (Figure S17, Supporting Information). This air sensitivity was only exhibited by films that had been subjected to electric fields: the conductivity of virgin films of **1** was found to be independent of prior exposure to air. This observation indicates that poled **1** degrades upon exposure to air, implying that the resistive switching mechanism involves the generation and retention of air‐sensitive species. Such degradation might involve disruption of the polymer chains, as supported by NMR studies (Section S3.10, Supporting Information).

By raising the temperature of a poled device, the low‐conductivity state was recovered (Figure 2c): When a device that had been “written” at 60 V for 9.5 min was heated to 346 K for 1.5 h, under vacuum and no electrical bias, the initial low‐conductivity state was restored. This recovery was reversible; subsequent operation of the thermally reset device revealed no appreciable signs of sample degradation, reaching similar values of current density when repeating the full experiment (Figure 2c, inset).

Finally, we performed short consecutive probe and pole cycles on our devices. Through applying a series of 30 s poling cycles at 40 V, different conductivity states of the device were accessed (Figure 2d). The number of distinct conductivity states depended upon the duration of the pole cycle: Longer poling cycles brought the metallopolymer further toward its maximum conductivity at a given voltage. Subsequent operation at 60 V (Figure 2d) further increased the number of accessible states.

**Figure 3:**
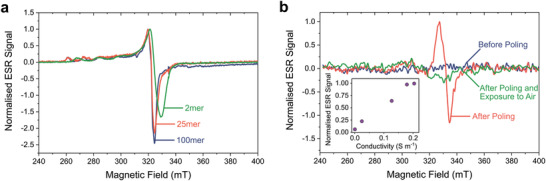
ESR evidence of Cu^II^ formation upon electrical poling. a) Solution‐state ESR measurements of oligomers of **1** with 2, 25, and 100 repeat units recorded in DMSO at 100 K under N_2_. b) In situ ESR measurements of an in‐plane device containing 50‐repeat‐unit metallopolymer **1** being poled under N_2_ at 300 K. The device consisted of two gold electrodes deposited on quartz, separated by a distance of 100 µm and with a width of 24.3 mm, on top of which was deposited a 50 nm film of **1**. The inset shows the normalized ESR signal as a function of conductivity recorded at the end of each poling step. Electrical characterization and description of the experimental setup are provided in Section S3.8.2, Supporting Information.

We propose that the mechanism of resistive switching within **1** is related to a change in the oxidation states of the linear array of copper centers. Traces of Cu^II^ in **1** that had not been poled were observed by low‐temperature ESR of **1** in frozen DMSO solution (**Figure** **3**a). In the frozen‐solution spectra, the presence of a low‐field quartet, paired with *g*‐values ranging from *g*_⊥_ = 2.07–2.08, was consistent with a population of Cu^II^ (*I* = 3/2) possessing axial symmetry and a $d_{x^{2}‐y^{2}}$ singly occupied molecular orbital (SOMO) (see Table S1, Supporting Information).^[^ ^30^, ^31^ ^]^

In the pristine (unpoled) state of **1**, the location of the Cu^II^ sites within the polymer core is thus fixed. This interpretation is supported by the observation of a low‐field quartet (Figure 3a), which implies hyperfine coupling of the unpaired electron to a single Cu nucleus.^[^ ^30^ ^]^ Shorter oligomers were found to contain a higher proportion of Cu^II^, ranging from ≈21% Cu^II^ in a 2mer to ≈4% in a 100mer (Figure 3a and Table S1, Supporting Information). These data suggested that, in the unpoled state, Cu^II^ is spontaneously generated in small amounts. The inverse proportionality between the amount of Cu^II^ and polymer length indicates that Cu^II^ may reside at the termini of **1**, where greater ligand flexibility facilitates reconfiguration^[^ ^32^ ^]^ of the ends of the helix to satisfy the square‐planar coordination geometry preference of Cu^II^.^[^ ^33^ ^]^ No intervalence charge transfer (IVCT) bands were observed in the near‐infrared spectral region of a pristine 50mer of **1**, which would have indicated the migration of discrete Cu^II^ centers along the chain.^[^ ^34^ ^]^ We hypothesize that the charges of the Cu^II^ sites at the termini of pristine **1** are balanced by radical anions hosted by the π* orbitals of the conjugated organic ligands in the ground state. Supporting this hypothesis, UV–vis absorption spectra of metallopolymer **1** displayed a low intensity absorption band at 950 nm, consistent with the presence of quinoline‐type radical anions, as was also observed in solution‐state transient absorption measurements (Figure S10, Supporting Information).^[^ ^33^ ^]^

We then employed in situ ESR spectroscopy to study the effects of poling on the oxidation state of the copper chain of metallopolymer **1** at 300 K. In‐plane devices based on the structure shown in Figure 1c, but with an inter‐electrode separation of 100 µm, were suspended under nitrogen in an air‐tight ESR tube, which allowed the device to be poled and its ESR spectrum recorded without altering its position in the microwave cavity (Section S3.8.2, Supporting Information). The X‐band ESR spectrum did not reveal any ESR signal for the device in the unpoled state (Figure 3b), implying an absolute number of spins at room temperature below the detection limit of the instrument, estimated at 10^13^ spins: given the volume of sample and of the polymer repeat unit (see discussion below), this limit allows us to estimate that no more than ≈0.5% of the Cu centers are in the Cu^II^ state before poling at room temperature. We ascribe the lower density of Cu^II^ in the in situ device experiments than in the frozen solution described above to a more rigid configuration of the helix in the solid state,^[^ ^27^ ^]^ as well as to the lower temperature at which the measurements were performed. Following poling at 20 V and removal of the electric field across the device, a clear ESR signal appeared between 320 and 350 mT. Successive 5 min/20 V poling steps resulted in an increase of the maximum current density, and hence the conductivity, at the end of each step. The intensity of the ESR signal increased with the conductivity of the device (Figure 3b, inset). All ESR signal intensity was lost upon exposing the device to air.

We attribute the ESR signal observed in the poled devices to Cu^II^ in the polymer chain. This assignment is in agreement with previous studies on Cu^II^ arrays in artificial DNA^[^ ^2^ ^]^ and with the ESR spectrum of **1** in frozen solution (Figure 3a). From the intensity of the ESR signal in the poled device (Figure 3b) we estimate the presence of (8 ± 4)× 10^14^ Cu^II^ centers in the sample at maximum poling (5 min at 20 V repeated 5 times, reaching 0.2 S m^–1^ in these experiments, Figure S14, Supporting Information, and inset in Figure 3b). Based on the active volume of **1** in the device and the volume of the repeat unit (4.07 × 10^–28^ m^3^, determined from the crystal structure of a tetra‐copper oligomeric analogue of **1**)^[^ ^26^ ^]^, we calculate that this value corresponds to (27 ± 13.5)% of Cu^I^ in **1** converting to Cu^II^ with poling. In poled devices, the measured *g*‐value is *g*_⊥_ = 2.03, less than the value of 2.07–2.08 observed in frozen solutions and closer to the value for a free electron (*g*_e_ = 2.002). This suggests the presence of a $d_{z^{2}}$ based SOMO for Cu^II^,^[^ ^35^, ^36^ ^]^ where the *z*‐axis coincides with the copper chain.^[^ ^32^ ^]^ As explained in more detail below, this $d_{z^{2}}$ SOMO configuration reflects Jahn–Teller distortion upon oxidation of Cu^I^ to Cu^II^. We conclude that radicals on the organic strands of the metallopolymer were not the main contributors to the ESR signal because the measured *g*_⊥_ deviated further from 2.002 than typical *g*‐values of organic radicals, either free^[^ ^32^, ^37^ ^]^ or bound within Cu complexes.^[^ ^38^ ^]^ These results cannot differentiate whether all chains in the film are undergoing oxidation to the same extent, or if parts of the film are left unoxidized. The estimation of (27 ± 13.5)% given above should thus be taken as a lower limit for the maximum proportion of copper centers susceptible to oxidation in a single chain.

To further understand the evolution of the electronic structure of metallopolymer **1**, we carried out spectroelectrochemistry upon **1** in solution. Changes in optical absorption as the potential was increased are assigned to the oxidation of Cu^I^ to Cu^II^ (Figure S20, Supporting Information). The appearance and growth of a broad, low‐energy absorption band at ≈3000 nm was observed upon oxidation of **1**. This band was only generated in systems containing multiple copper centers (Figure S21, Supporting Information) and was assigned to IVCT among the metal centers at the core of **1**, consistent with motion of charges in the $d_{z^{2}}$ SOMO between copper centers.^[^ ^34^, ^39^ ^]^ We also found that longer chains required a higher applied potential to bring about Cu^I^ oxidation than short oligomers, and that this oxidation process was electrochemically irreversible (Figure S18, Supporting Information). We infer that longer helices undergo more structural reorganization in order to optimize binding of Cu^II^. We hypothesize this reorganization to involve a winding of the helix, as described below and in Section S3.11, Supporting Information.

## 3 Discussion

**Figure 4:**
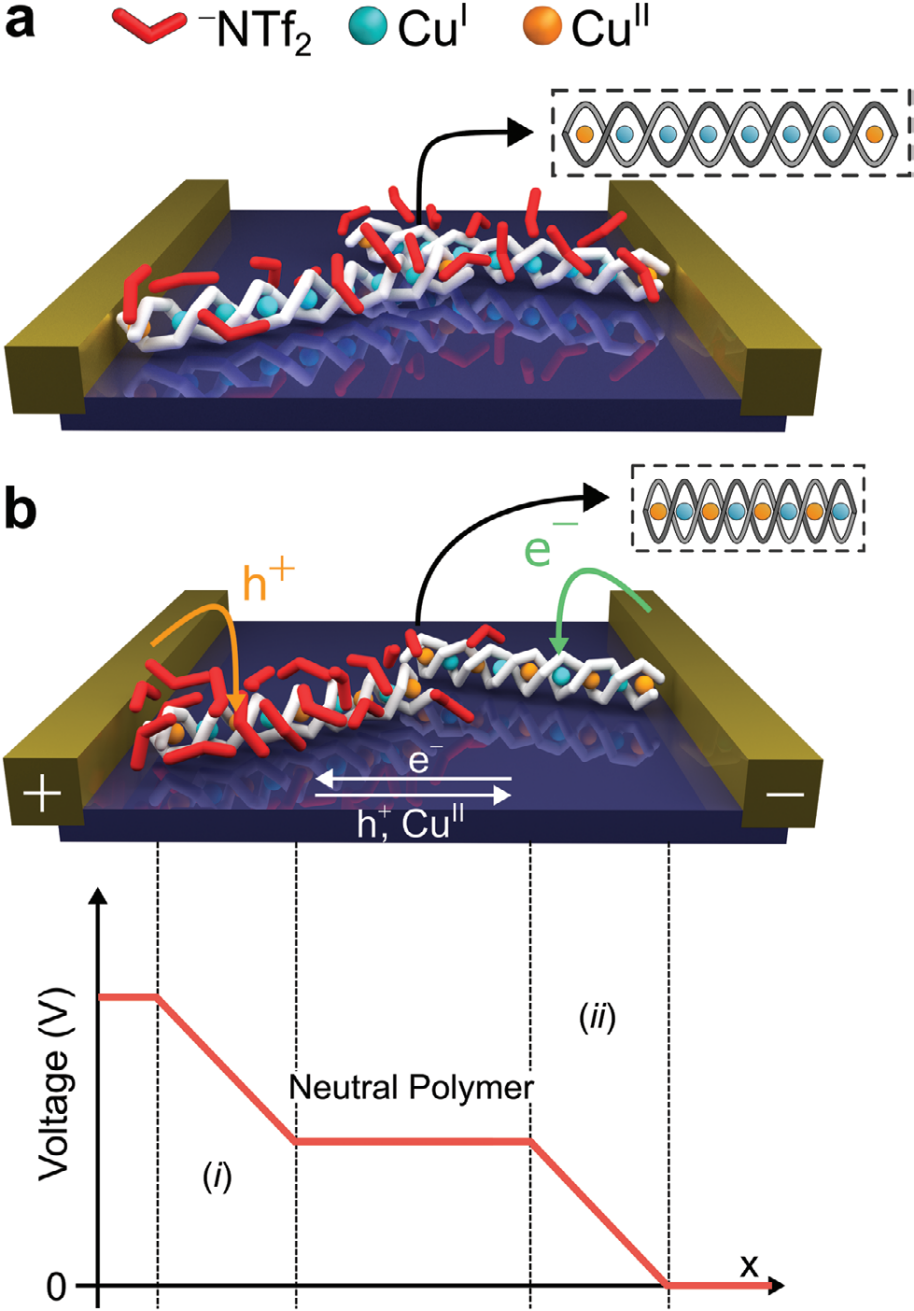
An idealized schematic representation of metallopolymer **1** in an in‐plane device; a real device contains chains with a distribution of orientations in the plane parallel to the substrate, with the majority of them not being in direct contact with the electrodes. a) The metallopolymer in its native state prior to poling, containing Cu^II^ only at the termini of the chain, is positioned between the electrodes. b) (top) Applying a voltage bias across the device triggers ^−^NTf_2_ migration; the potential drops across the surface‐charge‐dipole (SCD) regions formed at the electrodes (bottom), promoting oxidation of Cu^I^ to Cu^II^ at the SCD layer close to the positive electrode. Holes are injected into region *i*) while electrons are injected into region *ii*). (^‐^NTf_2_—red, Cu^I^—cyan, Cu^II^—orange)

Based on the results presented above, we propose the following mechanism to describe the resistive switching observed in metallopolymer **1**. In its pristine state, **1** contains a core of Cu^I^ atoms with Cu^II^ localized at its termini (**Figure** **4**a). This Cu^II^ is charge‐balanced and stabilized by the presence of radical anionic charge on the ligand strands.^[^ ^40^, ^41^ ^]^ When a voltage bias is applied, the ^–^NTf_2_ counteranions of **1** migrate, establishing SCD layers at each electrode (Figure 4b) that facilitate the injection of holes and electrons from the electrodes into **1**. The creation of these SCD layers at the electrodes manifests in the hysteresis observed in Figure 1e. The electric field at the positive electrode is strong enough to oxidize Cu^I^ to Cu^II^, with the latter drifting away from the electrode and along the copper chain via IVCT. As the hole migrates away from the electrode along the metallopolymer chain, there is further oxidation of Cu^I^ until the process becomes energetically unfavorable at the potential applied across the device. We hypothesize that counter anion migration is a required first step to produce the SCD layers that assist oxidation in our present devices, where the inter‐electrode separation is large, but that such migration would not be necessary in devices with smaller inter‐electrode distances.

The increased proportion of Cu^II^ in metallopolymer **1** is accompanied by an increase in the intensity of an ESR signal that is assigned to a $d_{z^{2}}$ SOMO, indicating that the degeneracy of the $d_{z^{2}}$ and $d_{x^{2}‐y^{2}}$ orbitals has been lifted during the oxidation process. In helicates composed of metal ions that adopt a tetrahedral coordination environment, a Jahn–Teller distortion can lift this degeneracy. For these molecules, two types of Jahn–Teller distortion are possible: A helical extension or a helical compression.^[^ ^33^, ^42^ ^]^ A helical extension would coincide with an increased separation of the copper ions in the chain, thus lowering the energy of the $d_{z^{2}}$ orbital with respect to that of the $d_{x^{2}‐y^{2}}$ orbital. In the case of an extension of a helicate containing d^9^ Cu^II^, the SOMO is therefore a $d_{x^{2}‐y^{2}}$ orbital and the *g‐*value obtained from ESR measurements must differ substantially from that of the free electron *g_e_* = 2.002. This was the case for the Cu^II^ ions at the termini of the metallopolymers in an unpoled state (*g*_⊥_ = 2.07–2.08, Figure 3a). In contrast, helical compression affords a reduced Cu^…^Cu separation and a more planar arrangement of the ligands around the metal center. This compression raises the energy of the $d_{z^{2}}$ orbital with respect to that of the $d_{x^{2}‐y^{2}}$, thus resulting in a $d_{z^{2}}$ SOMO for the Cu^II^ ions, and promotes a coordination environment closer to the square planar preference of Cu^II^.^[^ ^35^, ^36^, ^43^ ^]^ The shortened Cu^…^Cu distances further improves the orbital overlap between adjacent copper centers.^[^ ^42^ ^]^ Our ESR measurements (Figure 3b) show an increase in the $d_{z^{2}}$ character of the SOMO upon electrical poling, with a *g*‐value approaching *g*_e_ (*g*_⊥_ = 2.03), thus consistent with the oxidation process causing the helix to compress. The compression of the helix and the ability of the ligands of **1** to support additional charge^[^ ^40^, ^41^ ^]^ result in a metastable state, where re‐reduction is prevented and long‐lived charge‐separation occurs. To return to the initial higher‐resistance state, the activation barrier to helix‐extension must be overcome,^[^ ^33^ ^]^ which happens more rapidly when the material is heated.

We conclude that the linearly arrayed $d_{z^{2}}$ orbitals form a partly filled band that supports charge transport along the Cu core of **1**. Poled devices thus contain conductive channels consisting of partly oxidized double‐helical metallopolymer chains. To further test the role of the copper chain in charge transport, we characterized diodes comprising metallopolymer **1** sandwiched between two hole‐injecting contacts (ITO/PEDOT:PSS and MoO_3_/Au). Since the helical chains lie in the plane of the substrate, these devices probe charge transport in a direction perpendicular to them. At electric fields comparable with those used in the in‐plane devices, we observed no hysteresis and no resistive switching in the vertical devices (Section S3.9, Supporting Information). Furthermore, we found that in this configuration holes are transported via inter‐chain hopping, with the copper array not being involved in charge transport. These results confirmed that the resistive switching observed in in‐plane devices involves a change in charge transport behavior along the copper array and, importantly, that the organic ligand is effective in shielding the copper arrays from one another.

The inference that charge transport in the poled polymer happens along the oxidized copper core is further supported by comparison with **2**, a congener of **1** in which Ag^I^ was used in place of Cu^I^. Polymer **2** has the same structure as **1**, with helically wrapped ligand strands surrounding a core of Ag^I^ ions (Section 2, Supporting Information). The d^9^ Ag^II^ state is higher in energy than d^9^ Cu^II^.^[^ ^44^ ^]^ Polymer **2** showed 4–5 orders of magnitude lower conductivity than **1** (Figure S9, Supporting Information) and we concluded that poling produced no Ag^II^ within **2**. Finally, DFT calculations on dicopper(I) and disilver(I) model oligomers showed the HOMO residing principally on copper in the former case, but mostly on the organic ligands, and only minimally on silver, in the latter case (Section 3.12, Supporting Information).

As noted above, fully switched devices exhibit bulk conductivities of 2 S m^–1^ (Section 4.1, Supporting Information), but this value may be subject to future optimization. This conductivity is comparable to values reported for such one‐dimensional conductors as Krogmann^[^ ^18^ ^]^ and Magnus salts,^[^ ^19^, ^22^ ^]^ the conductivity of which has been measured on single crystals. Our study, in contrast, probed the bulk behavior of relatively disordered, solution‐processed films. In such films, the current flow is impeded by morphological traps and by the end groups of the metallopolymer, which may act as electronic traps; conduction between the termini of metallopolymers is inferred to take place by tunneling, a process that is enhanced upon formation of a partially filled band. Furthermore and as mentioned above, some of the chains may not have been fully converted into their oxidized, highest‐conductivity, state. Single chains or crystals of polymer **1**, oriented parallel to the electric field, may ultimately display higher conductivities. This orientation might be achieved by preparing the thin film in the presence of an electric field. Studies into the reorientation of the metallopolymer bundles when subjected to an electric field require experimental techniques to prevent exposing a poled device to air, the development of which is currently underway in our laboratories.

The observation of high electrical conductivity in nominally Cu^I^ materials has previously been associated with disproportionation to Cu and Cu^II^, whereby external stimuli such as electrical bias or high pressure generate conductive filaments of metallic copper.^[^ ^45^, ^46^ ^]^ We conclude that such irreversible disproportionation is not taking place in **1**, as indicated in the reversibility of the voltage‐driven increase in electrical conductivity (Figure 2c). This observation indicates that the polymer chain remains intact upon transition to higher‐conductivity states. In contrast, we infer that exposure of sample **1** to air in the low‐resistance state results in irreversible oxidation of the negatively‐charged ligand, leading to sample degradation as the Cu^II^ centers repel each other coulombically (Section 3.10, Supporting Information).

## 4 Conclusions

We have demonstrated that a linear array of copper atoms, constructed by self‐assembly of helical metallopolymers, can be made conductive upon oxidation via an externally applied electric field. A large proportion (≈25%) of copper ions in the array are oxidized from Cu^I^ to Cu^II^, a change that is accommodated by a physical “winding” distortion of the helix and by the organic ligand strand taking up anionic charge. The resulting change in electrical conductivity of the polymer can be retained over years, prevented from reversion to the initial state by the distortion of the helix. The organic strands also isolate the molecular wire from other adjacent chains in thin solid films, allowing the properties of isolated atomic chains to be exploited in bulk thin films. These copper helical metallopolymers thus represent an easily accessible platform for the study and application of charge transport at the nanoscale, with access to fine‐grained control over the structure–property relations.

## Conflict of Interest

The authors declare no conflict of interest.

## Author Contributions

J.L.G. and D.D.N. contributed equally to this work. J.L.G. synthesized the materials and characterized them by CD, CV, DLS, NMR, UV–vis, AFM and TEM. J.L.G. and D.D.N. performed all the experiments and analyzed all the data. E.W.E. and W.K.M. helped with the ESR measurements on frozen solutions. E.W.E. fitted the ESR traces of frozen solutions to extract *g*‐values and number of spins, performed the DFT calculations and helped with ultrafast spectroscopy. S.P.S. and J.T.D. fabricated the patterned substrates for in‐plane devices. S.P.S. helped with preliminary electrical characterization of in‐plane devices. S.S. fabricated the substrate for in situ ESR, helped with the measurement, and fitted the ESR traces to extract *g*‐value and number of spins. A.P. helped with the synthesis and characterization of the silver metallopolymer. D.D.N. conceived the project. D.D.N. and J.R.N. supervised the research. J.L.G., D.D.N., R.H.F., and J.R.N. wrote the manuscript, with input from all the authors.

## Supporting information

Supporting Information

## Data Availability

The data that support the findings of this study are available from the corresponding authors upon reasonable request.
