# Supplementary material for: Electrically Induced Mixed Valence Increases the Conductivity of Copper Helical Metallopolymers
Source: Adv Mater. 2021 May 6;33(24):2100403. doi: 10.1002/adma.202100403 (PMC11468940; doi:10.1002/adma.202100403)
Supplement: Supplementary file 1 — Supporting Information [file ADMA-33-2100403-s001.pdf]

© 2021 Wiley-VCH GmbH

ADVANCED MATERIALS

Supporting Information

for *Adv. Mater.,* DOI: 10.1002/adma.202100403

Electrically Induced Mixed Valence Increases the Conductivity of Copper Helical Metallopolymers

Jake L. Greenfield, Daniele Di Nuzzo, Emrys W. Evans, Satyaprasad P. Senanayak, Sam Schott, Jason T. Deacon, Adele Peugeot, William K. Myers, Henning Sirringhaus, Richard H. Friend,* and Jonathan R. Nitschke**

Supporting Information

Electrically Induced Mixed Valence Increases the Conductivity of Copper Helical Metallopolymers

Jake L. Greenfield[†], Daniele Di Nuzzo[†], Emrys W. Evans, Satyaprasad P. Senanayak, Sam Schott, Jason T. Deacon, Adele Peugeot, William K. Myers, Henning Sirringhaus, Richard H. Friend*, Jonathan R. Nitschke**

J. L. Greenfield and D. Di Nuzzo contributed equally to this work

Contents

1 Materials and methods 3
1.1 Materials 3
1.2 Methods 3
1.3 Device fabrication 5
2 Chemical synthesis and characterisation 7
2.1 Preparation of metallopolymer **1** 7
2.2 Preparation of metallopolymer **2** 7
2.3 Assembly of model compound **3** 8
2.4 Characterisation of polymer **2** 8
2.5 Charge transport properties of subcomponents 11
3 Supporting experimental data 12
3.1 AFM of Thin Films of metallopolymer **1** 12
3.2 TEM of semi-crystalline aggregates 13
3.3 Log-log plot of current density versus voltage of metallopolymer **1** 14
3.4 Effect of voltage polarity on current density 15
3.5 Projected retention of low resistance state 16
3.6 Charge transport properties of silver-based metallopolymer **2** 17
3.7 Evidence of charged ligands in the ground state 18
3.8 ESR studies 19
3.8.1 Solution-state ESR spectroscopy of **1** 19
3.8.2 In-situ ESR measurements on **1** 20
3.9 Out-of-plane charge transport in vertical devices of **1** 22
3.10 Degradation of poled **1** in air 24
3.11 Electrochemistry of metallopolymer **1** 26
3.12 DFT and TD-DFT calculations 27
3.13 Spectroelectrochemistry of metallopolymer **1** 29
3.14 Control measurements for spectroelectrochemical study 30
4 Calculations 31
4.1 Calculation of conductivities 31
4.2 Calculation of number of spins (ESR measurements) 31
5 References 32

1 Materials and methods

1.1 Materials

Cu(MeCN$_4$)NTf$_2$ was prepared following an adapted literature procedure,[1] and its purity checked by elemental analysis and ^{19}F NMR spectroscopy. AgNTf$_2$ was obtained from commercial suppliers and stored in the absence of light. All other reagents and solvents were purchased from commercial suppliers unless specified. 0.2 µm PTFE syringe filters were supplied by Whatman. Solvents used for assembly reactions were dried and degassed prior to use.

1.2 Methods

NMR spectra were recorded at 298 K using a Bruker AvanceIII HD Smart Probe 500 MHz spectrometer, automatically tuned and matched to the correct operating frequencies. The ^{1}H NMR measurements performed on films of **1** recovered from a poled device were carried out on a Bruker 500 MHz TCI cryoprobe spectrometer. TopSpin 3.5 and Mestrenova 11.0.4 were used to apply phase and baseline corrections. ^{1}H and ^{13}C NMR spectra were referenced to the residual solvent peak.

UV-vis measurements were performed on a Cary 5000 UV-vis-NIR spectrophotometer. Quartz cuvettes of either 1 mm or 10 mm path lengths were employed. For measurements of thin films, Spectrosil® substrates and ITO patterned glass substrates were employed. All solution-state measurements were temperature controlled and performed at 298 K (unless stated), maintained with a Peltier controller. Spectra were recorded in double beam mode, using only the front analyte beam to record spectra and leaving the rear beam open to air. A background spectrum containing only the chosen solvent (or blank substrate for solid-state measurements) was recorded prior to measuring samples. This background was subtracted from the sample data. Solutions for the UV-vis measurements were made using HPLC grade, dry, degassed solvents.

Circular Dichroism (CD) was performed on an Applied-Photophysics Chirascan qCD spectrometer using a 1 mm path-length cuvette. Experiments were recorded at 298 K, maintained with a Peltier temperature control. Measurements were background subtracted from blank solvent in an identical cuvette. The sample concentrations were adjusted to maintain a HV below 700 Volts. A minimum sample integration time of 1 second was used. The data was smoothed using a 5 pts Savitzky-Golay algorithm and the residuals checked for distortions. Solutions for the CD measurements were made using dry degassed solvents

and filtered through 0.2 µm PTFE membrane filters. No signs of linear dichroism or artefacts related to the self-assembly were observed.[2]

Dynamic Light Scattering (DLS) samples were prepared by preparing a solution of 2 mg/mL of polymer in MeCN, which was subsequently filtered through a 0.2 µm PTFE membrane filter. DLS spectra were recorded on a Malvern Zetasizer Nano S at 20 °C with a 633 nm laser. The sample was left to equilibrate at 20 °C for 120 s before measurement. A typical DLS experiment is comprised of at least 3 runs. In each run, at least 25 scans were taken, each elapsing for 10 s. The scan data was averaged by the Zetasizer software.[3] Each run was subsequently averaged.

TEM imaging was performed with a JEOL JEM-3010 (point resolution 1.7 **Å**) electron microscope operated at 300 kV. Solutions of polymer **1** (1 mL, 30 mg/mL in MeCN) were left to evaporate over the period of 3 days to afford a flat film. The solid sample was then collected, ground using a pestle and mortar and deposited on Cu grids (400 mesh) with a holey carbon supporting film.

Atomic Force Microscopy (AFM) AFM micrographs were obtained using an ANASYS Instruments nanoIR2. The AFM was operated in tapping mode with AN2-200 probes. Scan rates of 0.5 Hz were employed. Profile analysis was performed using Analysis Studio (3.14.6478), while statistical quantities (domain size) were collated based on the length of the longest diameter. A sample size of 40 was adopted. The material was deposited via spin-coating onto clean silicon dioxide substrates, analogous to the method employed for fabrication of the in-plane devices.

Solution-state cyclic voltammetry (CV) was performed using a BioLogic SP-150 potentiostat. The CV setup consisted of a glassy carbon working electrode, a Pt wire auxiliary electrode and an Ag/Ag^+ quasi-reference electrode. All measurements were performed under an Ar atmosphere and each measurement was referenced to an internal reference, Fc/Fc^+. A 0.1 M $TBANTf_2$/MeCN (TBA = $n$$Bu_4N^+$) electrolyte was used for cyclic voltammetry measurements while a 0.5 M $TBANTf_2$/MeCN electrolyte was used for spectroelectrochemical studies. Typical measurement parameters for cyclic voltammetry consisted of scan rates in the range of 25-400 mV s^{-1}. Spectroelectrochemistry was performed in an OTTLE cell comprised of a platinum gauze working electrode, platinum wire counter electrode, and an Ag/AgCl pseudo-reference electrode. Measurements were performed at 298 K and UV-vis absorption spectra were collected on a Cary 5000 UV-vis-NIR spectrophotometer. Samples were prepared in the absence of oxygen and water and the sample chamber was sealed with PTFE plugs.

Photoexcitation was achieved in the transient absorption (TA) measurements using a 400 nm pump by frequency doubling the 800 nm Ti:sapphire fundamental with a BBO (beta barium

borate) crystal. The probe light was generated by home-built broadband non-collinear optical parametric amplifiers (NOPAs) using the 800 nm output of a commercially available Ti:sapphire amplifier (Spectra Physics Solstice Ace). The delay between probe and pump pulses was varied using a Stanford DG645 delay generator. The transmitted probe pulses were collected with a silicon dual-line array detector that was driven and read out by a custom-built board from Stresing Entwicklungsbüro.

X-ray photoelectron spectroscopy was performed on Si/SiO_2 (300 nm) substrates using a Thermo Fisher ESCALAB 250Xi instrument (ultra-high vacuum).

ESR experiments on helical metallopolymers in DMSO were recorded on a Bruker EMX X-band CW-ESR spectrometer. The helical metallopolymers employed in these ESR studies were prepared in a N_2 glovebox, and transferred into ESR tubes under a N_2 atmosphere. The tubes were sealed air-tight prior to removal from N_2 atmosphere. For in-situ ESR measurements on devices, please refer to section 3.8.2 of the Supporting Information.

1.3 Device fabrication

All substrates were cleaned via bath sonication in acetone (15 min) followed by isopropyl alcohol (15 min) and finally plasma treated for 10 min. Films were deposited immediately after O_2 plasma treatment. For information on the devices employed for in-situ ESR experiments and on vertical devices for out-of-plane transport, please refer to sections 3.8.2 and 3.9 of the Supporting Information, respectively.

The in-plane devices were fabricated using Si/SiO_2 (300 nm) substrates with lithographically patterned Cr/Au electrodes (3 nm / 50 nm), with channel length and width of 10 and 700 µm respectively. No gate bias was applied in the measurements unless otherwise stated and the doped silicon layer played no role in the measurements, as it was completely insulated from the measurement circuit. The self-assembled helical metallopolymer (20 mg/mL in MeCN) was deposited directly onto the substrate by spin coating (2000 rpm, 2000 rpm/s acceleration for 2 min, followed by an additional step at 6000 rpm, 6000 rpm/s acceleration for 1 minute), yielding 50 nm thick films. Given film thickness, channel length and channel width, the cross-sectional area of the device used to calculate the current density is 3.5×10^{-11} m^2. The electrical characterisation was performed under vacuum (10^{-5} Bar) and at a controlled temperature in a probe station equipped with a semiconductor parameter analyser (Agilent 4155C). The chamber containing samples was evacuated for 1 h at 10^{-5} Bar before the start of measurements, to remove O_2 and H_2O from the polymer films in the devices. The patterned substrates were washed and re-used several times, with the polymers showing the same electrical behaviour each time: this observation rules out any possible substrate degradation effect as the cause of the observed changes in current. Current-voltage

characteristics (Figure 1e main manuscript) were performed at different scan rates to highlight the transient and long-lasting effect of ion migration and resistive switching, respectively. Calculation of current densities was achieved by dividing the current by the devices cross-sectional area (thickness of 50 nm and electrode length of 0.7 mm for Figure 1, 2 and 24.3 mm for the ESR studies in Figure 3 of the main manuscript).

2 Chemical synthesis and characterisation

2.1 Preparation of metallopolymer **1**

Polymer **1** was prepared according to previous procedures.[4,5] Briefly, under a N_2 atmosphere, **A** (20.0 mg, 116 µmol, 100 equiv) and **B** (0.4 mg, 2.3 µmol, 2 equiv) were added to a dry degassed solution of MeCN (1 mL). To this solution was added $Cu(MeCN)_4NTf_2$ (29.7 mg, 58.58 µmol, 50.5 equiv). This was left to stir under N_2 for 72 h. The resulting dark brown solution was stored under N_2 prior to use. The enantiopurity of **B**, employed as the (*R*)-enantiomer, engenders an excess of the *M* handedness of polymer **1**. The polymer possesses preferential head-to-tail regiochemistry.

2.2 Preparation of metallopolymer **2**

Ag^I polymers were prepared and subsequently characterised in an analogous way to previously reported Cu^I congeners. Although Ag^I is not prone to oxidation, self-assembly was carried out under a N_2 atmosphere in the absence of light and stored under identical conditions to **1** prior to use in experiments and characterisation.

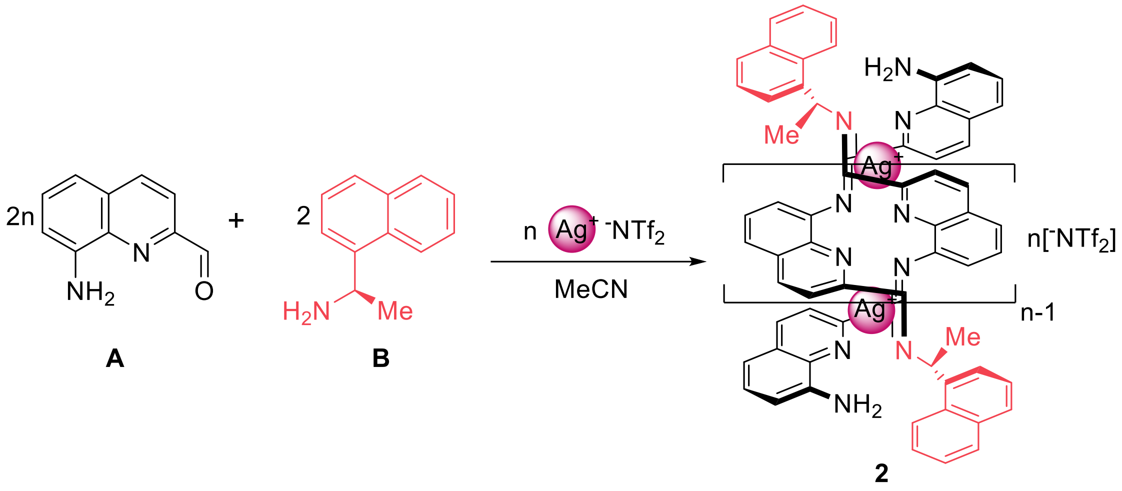

Under a N_2 atmosphere and in the absence of ambient light, **A** (20.0 mg, 116 µmol, 100 equiv) and **B** (0.4 mg, 2.3 µmol, 2 equiv) were added to a dry degassed solution of MeCN (1 mL). To this solution was added $AgNTf_2$ (22.7 mg, 58.58 µmol, 50.5 equiv). This was left to stir under N_2 for 72 h. The resulting red solution was stored under N_2 prior to use in an amber glass vial. The enantiopurity of **B**, employed as the (*R*)-enantiomer, engenders an excess of the *M* handedness of polymer **2**. The polymer possesses preferential head-to-tail regiochemistry.

2.3 Assembly of model compound **3**

Compound **3** was prepared so as to afford a model compound containing only 1 Cu^{I} ion between the two conjugated ligands. This model compound was employed in the control spectroelectrochemistry experiments presented in Figure S21.

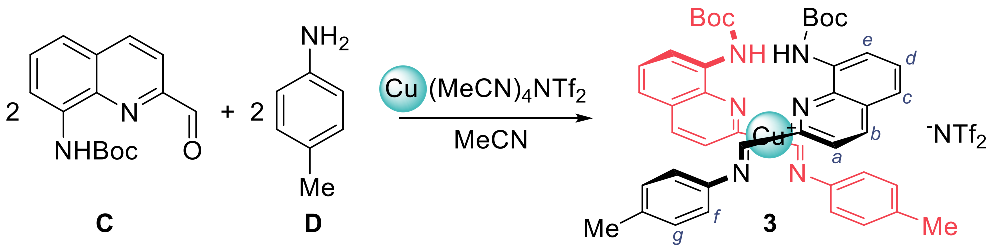

To dry, degassed MeCN (1 mL) was added tert-butyl (2-formylquinolin-8-yl)carbamate, **C**, (20.0 mg, 73.4 μmol, 2 equiv), *p*-toluidine, **D** (7.9 mg, 73.7 μmol, 2 equiv) and $Cu(MeCN)_4NTf_2$ (18.6 mg, 36.7 μmol, 1 equiv) under a N_2 atmosphere. The solution was left to stir at r.t. for 48 h under N_2. The product was isolated by removing the solvent in *vacuo* yielding a light brown solid, **3**, (37.9 mg, 97% yield). ^{1}H NMR (500 MHz, 298 K, CD_3CN): δ 8.92 (br s, 1H, NBoc***H***), 8.79 (s, 1H, NC***H***), 8.38 (d, *J* = 7.2 Hz, 1H, $\boldsymbol{H_e}$), 8.35 (d, *J* = 8.5 Hz, 1H, $\boldsymbol{H_a}$), 8.23 (d, *J* = 8.5 Hz, 1H, $\boldsymbol{H_b}$), 7.59 (dd, *J* = 8.0, 7.2 Hz, 1H, $\boldsymbol{H_d}$) 7.57 (d, *J* = 8.0 Hz, 1H, $\boldsymbol{H_c}$), 7.33 (d, *J* = 8.2 Hz, 2H, $\boldsymbol{H_f}$), 7.27 (d, *J* = 8.2 Hz, 2H, $\boldsymbol{H_g}$), 2.38 (s, 3H, Ar-C$\boldsymbol{H_3}$), 1.56 (s, 9H, C(C$\boldsymbol{H_3}$)$_3$); ^{13}C NMR (125.7 MHz, 298 K, CD_3CN) 160.2, 153.6, 153.5, 148.5, 138.7, 138.5, 136.2, 130.9, 130.0, 129.5, 122.4, 121.6, 120.4, 116.4, 81.4, 30.9, 28.5, 21.1; ESI-MS: *m/z* 785.23 [**3**-NTf_2]$^+$; HRMS (ESI-LTQ Orbitrap, MeCN) for [**3**-NTf_2]$^+$: *m/z* calcd: 785.2871; found 785.2857.

2.4 Characterisation of polymer **2**

The self-assembly of **A** around Ag^{I} behaves similar to the Cu^{I} analogue. Figure S1 and Figure S2 below show that the length of polymer **2** can be controlled in an analogous manner and that polymer **2** adopts the similar regiochemical-dependent growth mechanisms to polymer **1**.

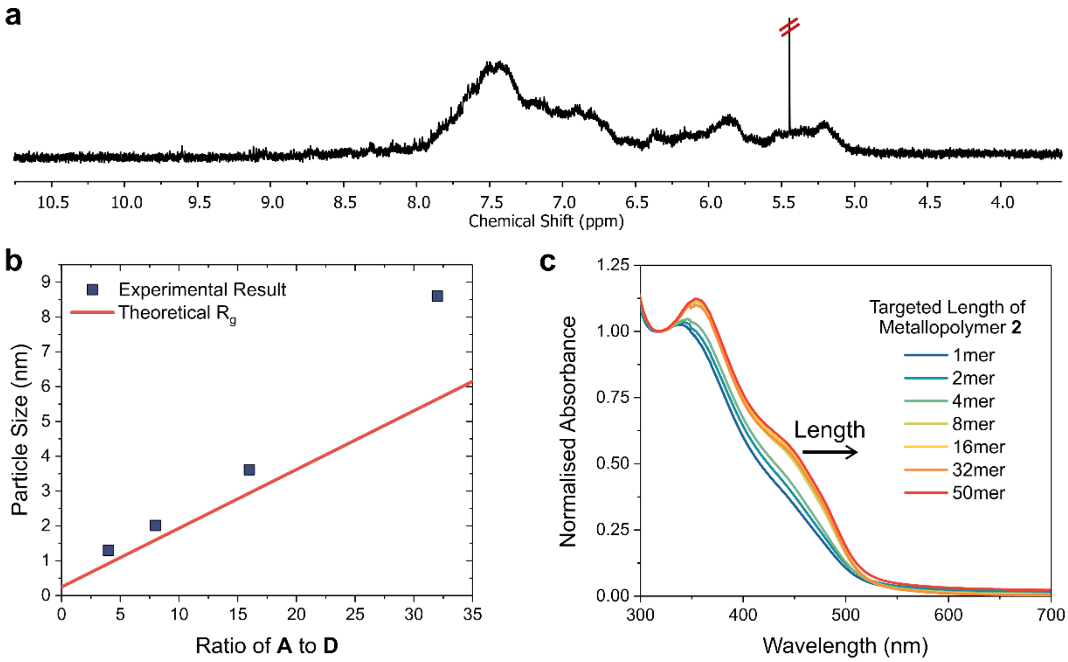

Figure S1. **a**, The ^{1}H NMR spectrum (500 MHz, 298 K, CD_3CN) of polymer **2** in dry degassed CD_3CN. The absence of the aldehyde signal at *ca.* 10 ppm of **A** indicates consumption of the monomer subcomponent, while the broad aromatic region is indicative of polymer formation.[4,5] **b**, The particle size (obtained from number% DLS plots) as a function of monomer **A** to end-cap *p*-toluidine, **D**.[4] The particle size tracks with the expected increase in the radius of gyration, R_g, that was calculated based on a 3mer of polymer **1**.[4] **c**, The UV-vis spectra of different length oligomers of **2**, ranging from a 1mer to 50mer. The spectral intensity was normalised at 317 nm. The lowest energy transition red-shifts for increasing polymer length, which we attribute to an increase in conjugation length and a decrease in band gap energy.

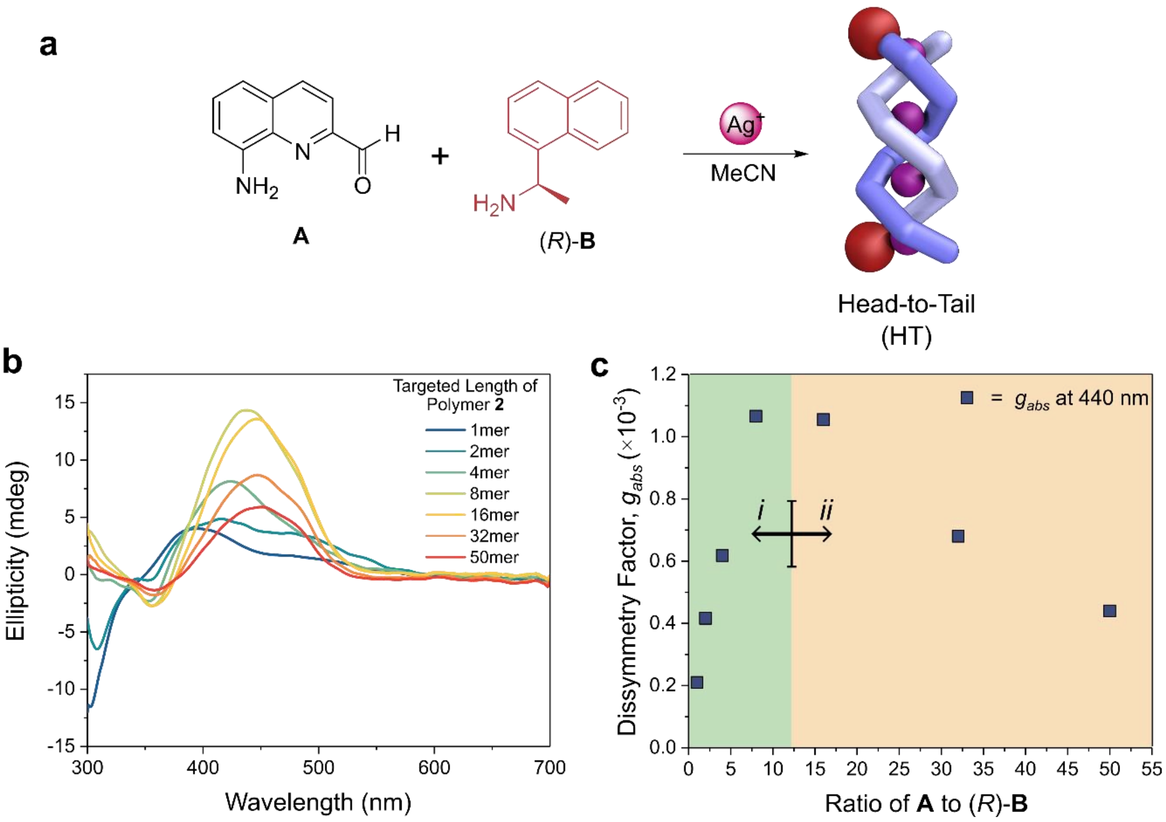

Figure S2. a, It has been shown previously[5] that end-group **B** can enforce a head-to-tail (HT) regiochemistry, respectively, for polymer **1**. It was envisaged that these rules apply to polymer **2**. **b,** The circular dichroism spectra of oligomers of **2** with a HT regiochemistry and ranging from a 1mer to a 50mer. Samples were prepared at a concentration of 20 mmol (with respect to **A**). A red-shift of the CD band at *ca.* 400-450 nm was observed, which we attributed to an increase in the conjugation length of the polymer. **c**, The plot of g_{abs} as a function of the stoichiometric ratio of monomer **A** (prepared at 20 mM in MeCN) to end-cap **B**. Here we observed two regimes, similar to the behaviour that was observed for the HT regioisomer of the Cu polymer.[5] The initial rise in g_{abs} (region i) is attributed to an increase in nucleation and intrinsic increase in g_{abs} as a function of length.[5] The decrease in g_{abs} in region (ii) is attributed to the lesser statistical likelihood of a polymer initiating from chiral amine (*R*)-**B**.

2.5 Charge transport properties of subcomponents

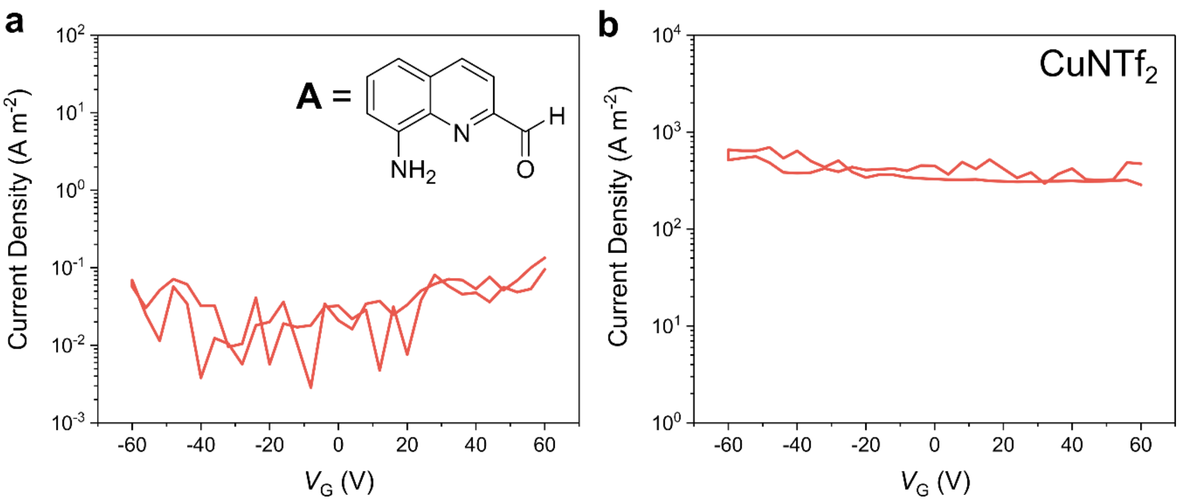

Figure S3. **a**, The performance of the monomer unit as a semiconductor was evaluated by recording the transfer characteristics in an in-plane device architecture (Figure 1c in main manuscript) operated as a bottom-gate bottom-contact Field-Effect Transistor, i.e. by scanning the gate voltage while applying a source-drain voltage of 60 V and measuring the current density between source and drain. A solution of monomer **A** (20 mg, 116 µmol) in 1 mL of DMSO was prepared. DMSO was selected as the solvent as the monomer exhibits limited solubility in MeCN in the absence of Cu^{I}. The transfer characteristics exhibit significantly low current density without any gate modulation indicating insulating behavior. **b**, Thin films comprised of $CuNTf_2$ were prepared *via* spin coating a solution of $CuNTf_2$ in MeCN (30 mg/mL). Here, the Cu^{I} salt showed poor conductivity.

3 Supporting experimental data

3.1 AFM of Thin Films of metallopolymer **1**

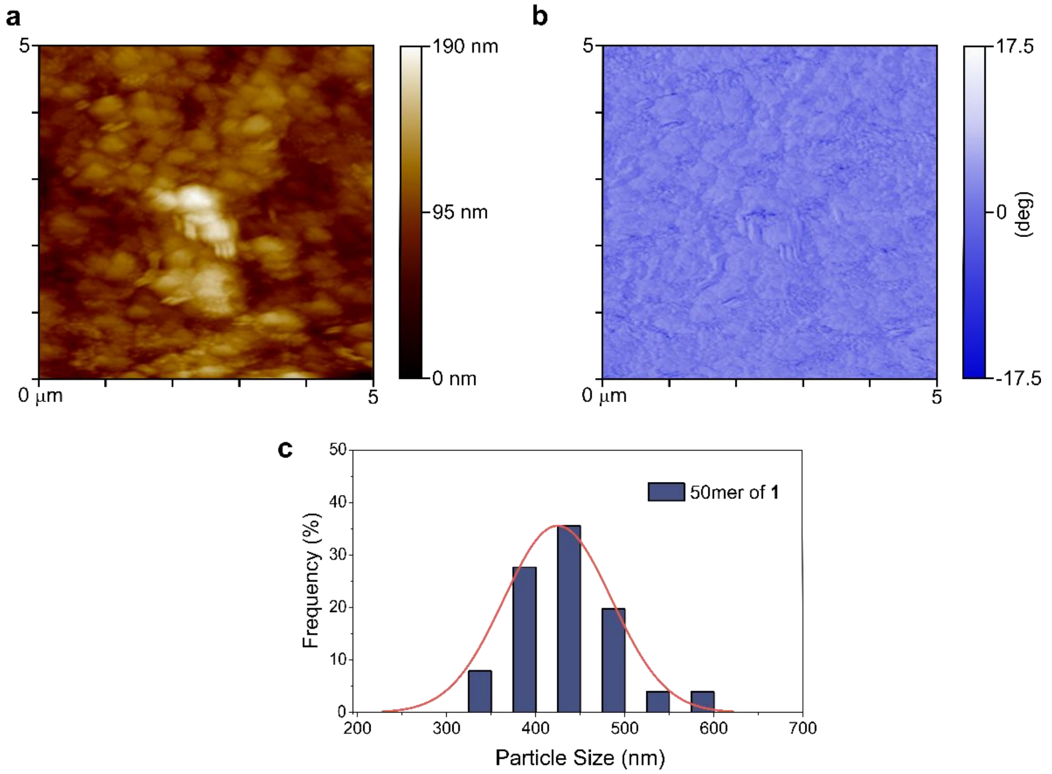

Figure S4. **a**, AFM height image of a thin film of metallopolymer **1**, obtained in tapping mode. This film was deposited following the procedure used to fabricate the in-plane device. **b**, The AFM phase image corresponding to that shown in panel **a**. **c**, The distribution of domain sizes observed under AFM for a 50mer of metallopolymer **1**, obtained using AFM. Data were obtained by measuring the longest axis of domains using a minimum statistical sample size of 40. A mean domain size of 420 nm (with a standard deviation of 60 nm) was obtained. The films used in this AFM study contain a 50mer metallopolymer exhibiting head-to-tail regiochemistry and a 52% excess of *M* helical handedness.

3.2 TEM of semi-crystalline aggregates

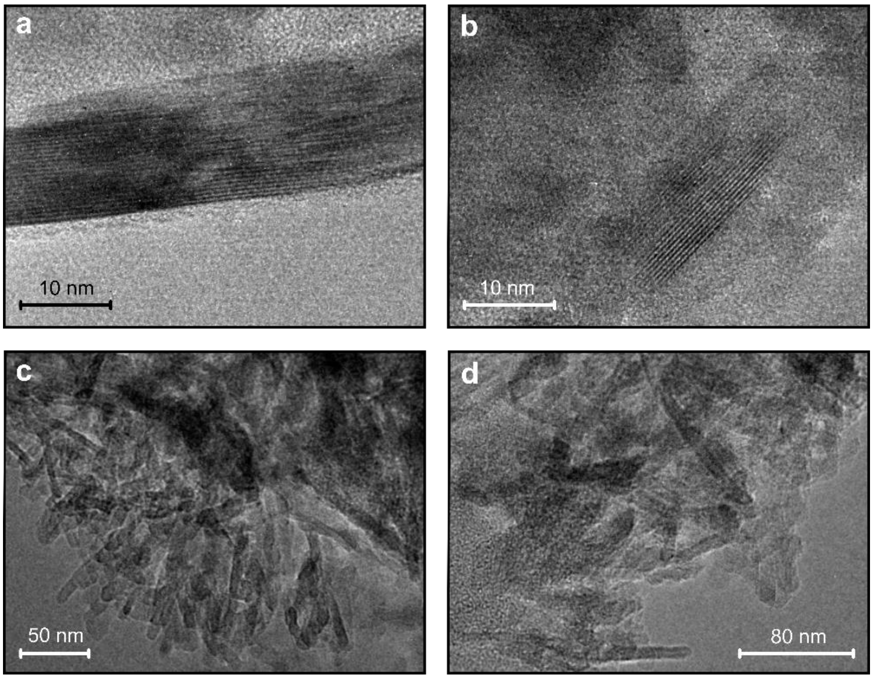

Figure S5. High-resolution TEM images showing high-contrast rod-like objects in films of **1**. **a**, Striations are observed running along the length of the rod with spacing consistent with the polymers adopting hexagonal close pack structure.[5] **b**, Rods of different length and aspect ratio were observed throughout the sample. Low-resolution TEM images **c**, and **d**, show the high-aspect ratio rods predominantly lying flat on the substrate.

3.3 Log-log plot of current density versus voltage of metallopolymer **1**

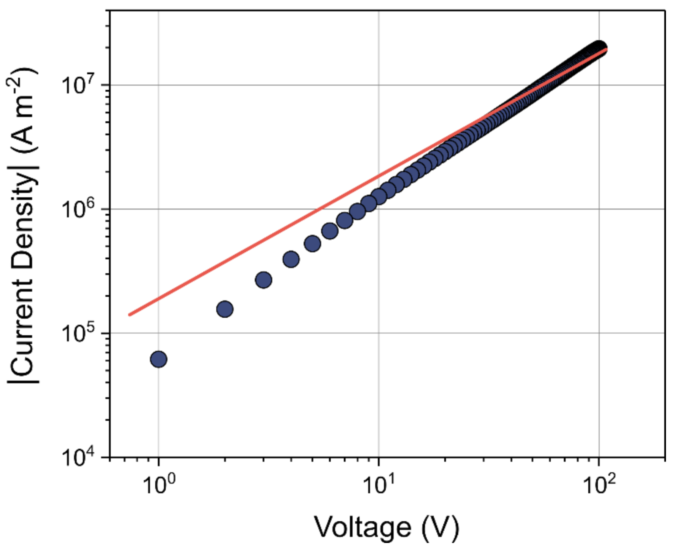

Figure S6. Log-log plot of the absolute current density versus voltage for metallopolymer **1** at slow scan rate, shown in Figure 1e of the main manuscript (scan 4). The red line has slope 1 in the log-log plot and hence corresponds to linear dependence of current density on voltage, i.e., ohmic conduction. The sub-linearity at voltages below *ca.* 50 V is attributed to a barrier of injection for charges, which is fully removed only once ion accumulation at the electrodes is complete (i.e. at higher voltages or after longer poling times).

3.4 Effect of voltage polarity on current density

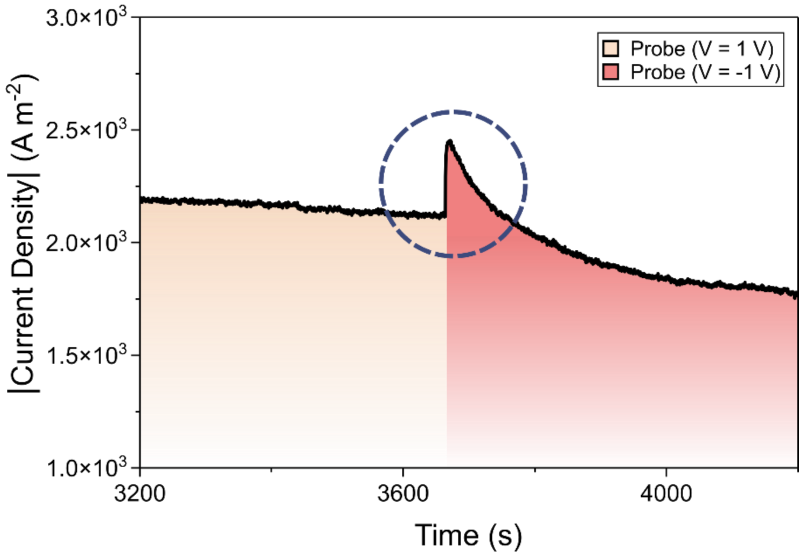

Figure S7. Data from Figure 2a in the main manuscript focusing on the small transient increase in the absolute current density as the polarity of the pole cycle is changed, highlighted by the blue circle.

Upon switching from 1 V to -1 V, we observed a small transient increase in current; integrating the transient current over a time of 800 s (after which the current decays with the same slope as before switching to -1 V) gives an estimated 2×10^{-5} charges displaced, corresponding to 6×10^{10} charges per m^3. We attribute this transient increase in current density to a redistribution of $^{-}NTf_2$ anions that were previously accumulated at the positive electrode. In the absence of ionic redistribution, the capacitance of the structure would in fact correspond to an accumulated charge on the order of 10^{-16} C, relating to a stored charge several orders of magnitude lower than what was observed here.

3.5 Projected retention of low resistance state

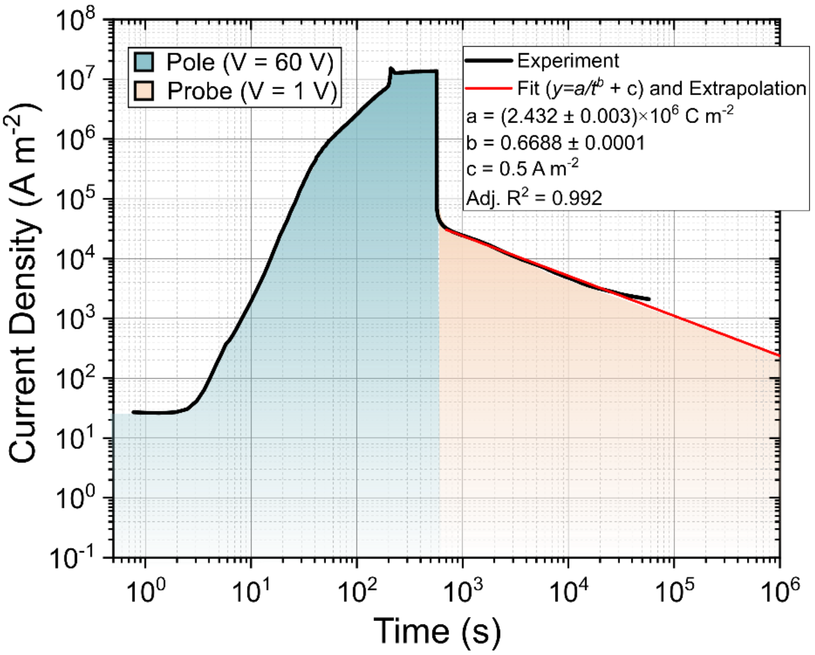

Figure S8. The projected retention time (memory) of the low resistance state for a device comprised of 50mer of metallopolymer **1** at 300K in vacuum. This is the same data shown in Figure 2b in the main manuscript (excluding the exposure to air). The curve was fitted to a power law function as indicated in the figure. The data selected for the fit were from t=700 s until the end of the scan (5.8×10^4 seconds, ≈16 hours); 700 seconds corresponds to 130 seconds after the voltage was switched to 1 V and was considered long enough to exclude the first, faster part of the decay determined by ionic redistribution lowering the charge injection efficiency. The decay curve is extrapolated using the fitted function, in order to estimate the retention time of information stored in the films. If this extrapolation reflected reality, information would be stored and read on a time scale of more than a decade.

3.6 Charge transport properties of silver-based metallopolymer **2**

The charge transport through the silver-based metallopolymer **2** was tested in in-plane devices (Figure 1c in main manuscript) operated as Field-Effect Transistors, by scanning the gate voltage while applying a source-drain voltage of 60 V and measuring the current density between source and drain. The measurements were conducted in vacuum and at 300 K. In this case the channel length and width are 5 and 700 µm respectively. The film thickness of **2** was 50 nm, obtained following the same coating procedure detailed in Section 1.3.1 above. A 50mer of **2** was used, possessing head-to-tail regiochemistry.

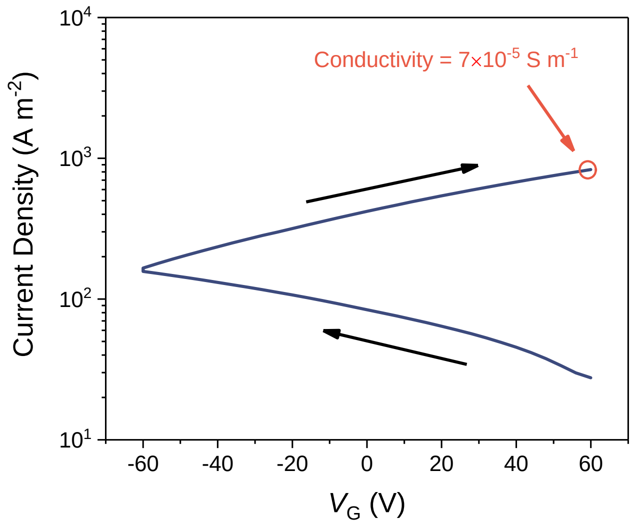

Figure S9. Transfer characteristic of an in-plane device comprising a layer of **2**, operated as Field-Effect Transistor. The arrows indicate the direction of scan: we attribute the observed hysteresis to progressive accumulation of ions at the source and drain facilitating injection of charges into the polymer. We use the current density at the end of the scan (indicated by a red circle) to estimate the bulk conductivity of the material, which amounts to 7 $\times 10^{-5}$ S m^{-1}. Note that this value is 4-5 orders of magnitude lower than the conductivities found in copper-based metallopolymer **1** reported in the main part of the manuscript.

3.7 Evidence of charged ligands in the ground state

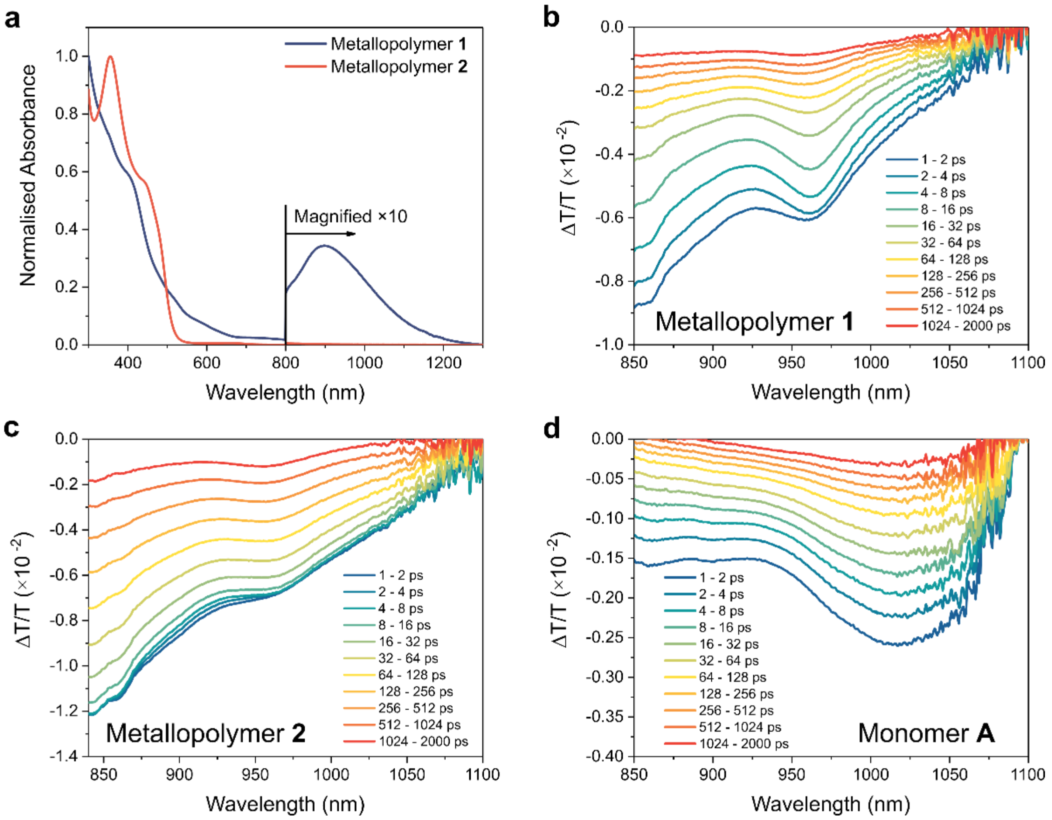

Figure S10. a, The UV-vis absorption spectra of metallopolymer **1** (blue) and its Ag^{I}-containing analogue **2** (red) in MeCN. Notably, **1** exhibited a MLCT band in the range 500-700 nm, whereas **2** did not. The intensity of the spectral range above 800 nm was magnified for clarity. **1** exhibited a band around 900 nm, attributed to the radical anion of the quinoline ligand, which is absent in **2**. **b**, The transient absorption spectra of **1** showing a photoinduced absorption (PIA) in the spectral range assigned to the ligand-based radical anion. **c**, The transient absorption spectra of **2**, showing a PIA between 900-1050 nm. **d**, The transient absorption spectra of **A** showing a PIA between 900-1050 nm. TD-DFT calculations (see Supporting Information section 4.1) predict the lowest energy transitions of **1** and **2** exhibiting MLCT and π-π* character respectively. In both cases, this results in the generation of a radical anion on the helical strand. Due to the wavelength of these PIAs, we infer the ground state of **1** to possess a radical anion on the helical strand, charge balanced by the presence of the Cu^{II} at the termini of the metallopolymer chain (Figure 3 in manuscript). The transient absorption measurements were performed in a sealed cuvette with the samples dissolved in dry degassed MeCN. An excitation wavelength of 400 nm was employed at 100 μJ/cm^2 fluence.

3.8 ESR studies

3.8.1 Solution-state ESR spectroscopy of **1**

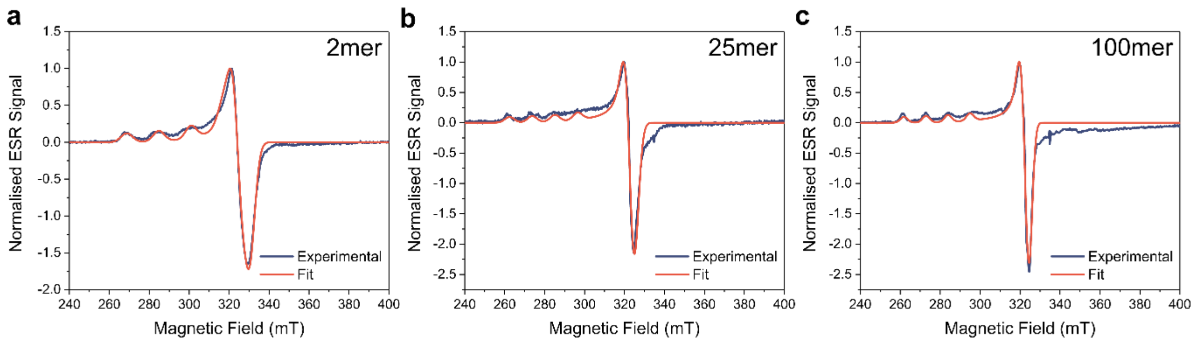

Figure S11. The X-band ESR spectra for **a**, 2mer **b**, 25mer and **c**, 100mer of polymer **1** in DMSO, recorded at 100 K (0.2 mT modulation amplitude). The four features at lower magnetic fields are indicative of the presence of Cu^{II} (S=1/2, I = 3/2). The spectra were fitted, with parameters from the fit detailed in Table S1.

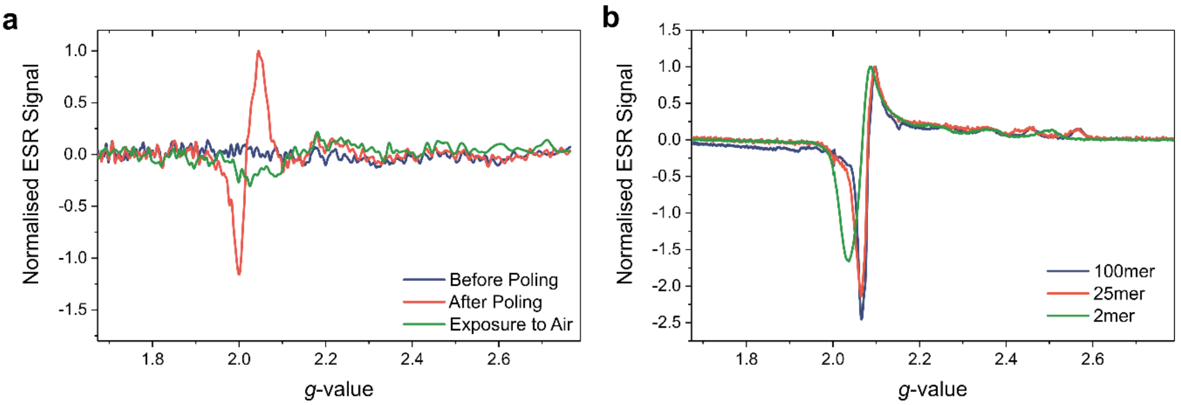

Figure S12. The normalised ESR intensity, as shown in Figure 3 in the main manuscript, plotted as a function of *g*-value. **a**, The in-situ ESR measurements performed on a device (shown in Figure S13) containing **1**. **b**, The solution state ESR measurements performed on oligomers of **1**.

Table S1. The difference in the percentage of Cu^{II} in different length oligomers at 100 K, as calculated using spin quantification. The g-values are presented, showing a low $g_{\perp}$ indicative of the SOMO showing $d_{x^2-y^2}$ character.[6] The results from in-situ ESR measurements at room temperature on a poled device (5 consecutive times at 20 V for 5 minutes each time) are also reported for comparison.

	% Cu^{II}	$g_{\perp}$	$g_{\parallel}$
2mer	21%	2.0656	2.2891
25mer	11%	2.0772	2.3975
100mer	4%	2.0806	2.4085
Poled Device (50mer)	27%	2.0167	n/a

3.8.2 In-situ ESR measurements on **1**

In-situ ESR measurements were performed at 300 K on a device using the experimental setup depicted in Figure S13 below. Thin films of a 50mer of **1** (unfiltered, enantiomerically enriched) were deposited on a 3 mm × 40 mm quartz glass substrate with gold electrodes by spin coating. We used interdigitated electrodes comprised of a Cr/Au bilayer (3 nm and 50 nm, respectively) which were lithographically patterned on top of the substrate. The distance between electrodes in this device was 100 μm, with a total electrode width of 24.3 mm; the electrode fingers were 50 μm large and the total active area was 2.45 mm × 20 mm. Contact pads for electrical connections are located at the top of the sample, about 10 mm away from the active area, to be placed outside the microwave cavity. Wires were connected to the electrodes which passed through an air-tight rubber seal. The substrate and wires were inserted into an ESR tube under an inert N_2 atmosphere and stoppered, affording an inert atmosphere inside the tube.

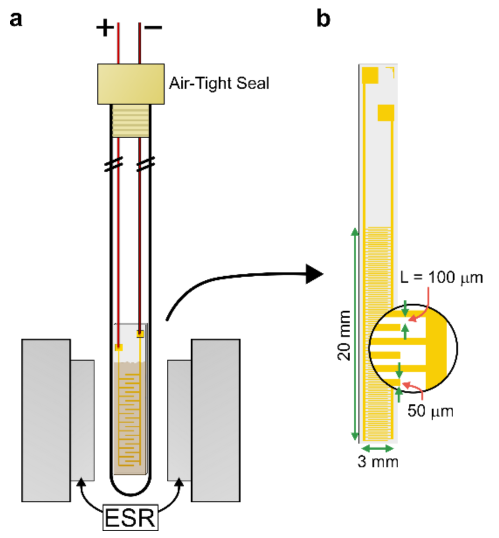

Figure S13. **a**, Schematic of the experimental setup for the in-situ ESR measurements. The substrate is stored and poled under a N_2 atmosphere. **b**, Representation of the substrate employed in this measurement.

Electron spin resonance (ESR) measurements were performed on a Bruker E500 X-band spectrometer with an ER 4122SHQE cavity at a microwave frequency of 9.368 GHz and a microwave power of 6.325 mW. The external magnetic field was modulated at 100 kHz with an amplitude of 1.5 mT and the spectra were recorded as first harmonics. All spectra were recorded at room temperature with an integration time of 120 ms per data point. The device was operated at either 10 or 20 V for a period of either 2 or 5 min, after which the sourcemeter was disconnected and the ESR measurement was recorded. The choice of 20 V was dictated by setup constraints and at the same time allowed to achieve resistive

switching at a slow enough rate to be probed via ESR. The absolute number of spins was estimated to be (8±4)×10^{14} from the magnetic susceptibility, as calculated by double integration of the first harmonic spectrum, by assuming a spin of S=1/2 and a Curie temperature dependence of the susceptibility.[7]

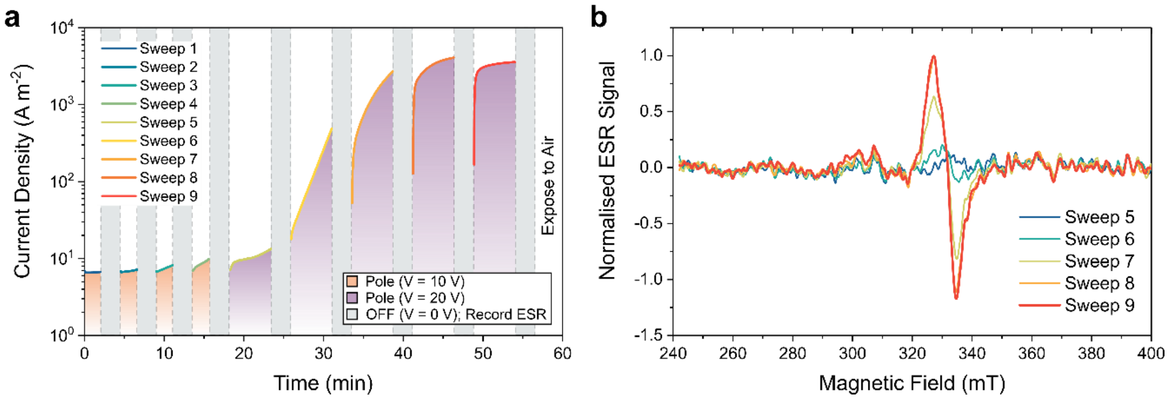

Figure S14. a, The plot of current density against time for a series of poling cycles and ESR measurements. The distance between electrodes in this device was 100 μm. A typical cycle consisted of a 2 or 5 min poling time (at either 10 or 20 V, orange and purple regions respectively) followed by a measurement of the ESR spectrum in the absence of an applied voltage (grey region). Sweeps 1-4 afforded only a small change in current density; no ESR signals were observed for these sweeps. Therefore, the poling voltage was increased to 20 V and applied for 5 min, noting a change in current density. **b**, The ESR spectra of sweeps 5-9 showing an increase in the ESR signal assigned to Cu^{II} (with a d_{z^2} SOMO). Panels **a** and **b** highlight that an increase in current density is accompanied by an increase in the intensity of the ESR signal.

3.9 Out-of-plane charge transport in vertical devices of **1**

A vertical device structure was employed to investigate the transport of holes in metallopolymer **1** in a direction perpendicular with respect to the plane in which the polymer chains lie ("out-of-plane", Figure S15a). The energy levels of the layers used in the vertical structure are shown in Figure S15b. The HOMO energy of metallopolymer **1** was obtained via cyclic voltammetry (Figure S18) and the LUMO was calculated by using the HOMO and the optical band gap.

Vertical devices were fabricated by spin coating PEDOT:PSS (Clevios P VP AL4083) in air (5000 rpm, 2000 rpm/s acceleration for 1 min) onto patterned indium tin oxide substrates (15 Ω, purchased from Colorado Concept Coatings). The PEDOT:PSS layer was subsequently annealed at 200 °C for 20 min under N_2 atmosphere. A 50 nm film of 50-mer of metallopolymer **1** was then spin coated on top of PEDOT:PSS. The devices were completed by thermal evaporation of MoO_3 (10 nm) and Au (100 nm) electrodes through a shadow mask. These devices were encapsulated with a glass cover and epoxy under N_2 atmosphere. The vertical devices were measured in the dark at 300 K, monitoring the injection of holes from the top contact (MoO_3/Au) into the semiconductor. Current Density-Voltage curves were recorded with a Keithley 2400 source meter.

The vertical devices displayed a clearly different electrical behaviour compared to the in-plane ones (Figure 1e, main manuscript). A double *J-V* scan is shown in Figure S15c, demonstrating that the device did not present neither hysteresis nor resistive switching at electric field comparable to those used with the in-plane devices (10^7 A m^{-1}). Furthermore, much lower current densities were reached compared to the fully switched in-plane devices: at 1 V, corresponding to an electric field of 2×10^7 V m^{-1} across the polymer film, metallopolymer **1** afforded a current density of 10^1 A m^{-2}, i.e. 6 orders of magnitude lower than in in-plane devices at 10^7 V m^{-1} (Figure S15 here and Figure 1, 2 in the main manuscript).

Furthermore, the vertical devices showed the typical current-voltage behaviour of a metal-insulator-metal structure, displaying space-charge limited current (Figure S15d). MoO_3 achieves ohmic injection into the semiconductors,[8] allowing to measure the zero-field hole mobilities by using the modified Mott-Gurney law, which incorporates a field-dependent mobility term β:

$$J(V) = \frac{9}{8}\varepsilon_0\varepsilon_r\mu_0\frac{V^2}{d^3}\exp\left(\beta\sqrt{\frac{V}{d}}\right)$$

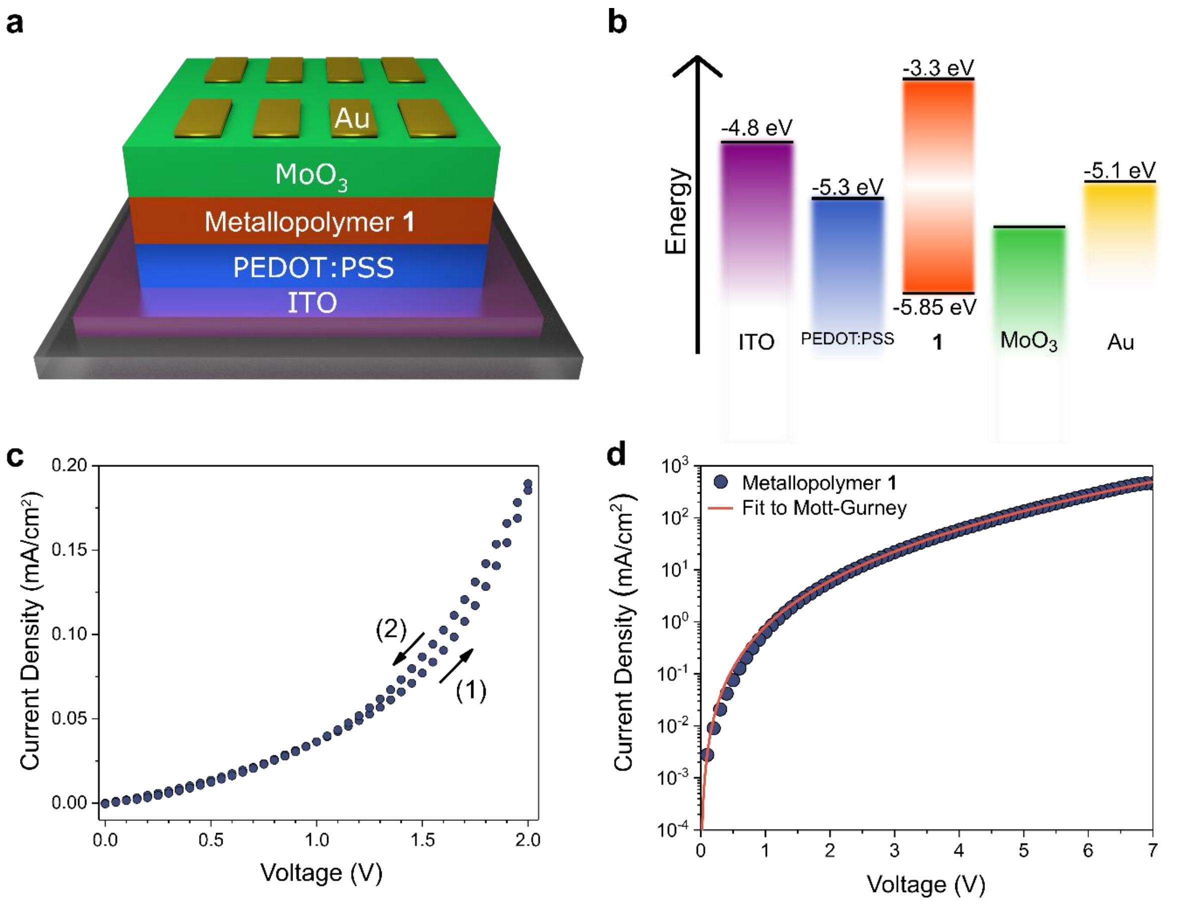

Figure S15. a, Schematic representation of the vertical device structure. **b**, Energy levels (in eV) of the components in the vertical device with respect to vacuum. **c,** Current density-voltage plot of metallopolymer **1** in the vertical device. The voltage range shown here was selected to be comparable with the electric field present in the in-plane device reported in the main manuscript. The arrows and numbering indicate the scan direction and scan order, respectively. **d**, The current density-voltage plot of **1** in the vertical device displaying space-charge limited current, and the fit of this data to the modified Mott-Gurney law. Values used in this fit are displayed in Table S2. Both panels c and d show hole injection from the top Au/MoO_3 contact into the semiconductor.

where μ_0 is the zero-field mobility, ε_0 and ε_r are the vacuum and relative permittivity, respectively, and *d* is the film thickness. By fitting the *J-V* curve in Figure S15d to the Mott-Gurney expression we obtain a hole mobility of 4.4×10^{-5} cm^2 $V^{-1}s^{-1}$. The results of the fit are summarised in Table S2 below. The hole mobility we find is typical for organic semiconductors.

In summary, the absence of hysteresis and the value of the hole mobility indicate that out-of-plane charge-transport proceeds via interchain hopping through the conjugated organic strands without involving the copper array. The copper array of each chain is thus isolated from the other chains by the organic ligand. These results confirm that resistive switching in this material must involve a change in conduction in the copper array.

Table S2. Fit parameters of the *J-V* curve of Figure S15d.

μ_0 (cm^2/Vs)	ε_r	β ($cm^{1/2}$ $V^{-1/2}$)	*d* (nm)	Adjusted R-square
$(4.4\pm0.1)\times10^{-5}$	5	6.5×10^{-3}	50	0.998

3.10 Degradation of poled **1** in air

Solution-state ^{1}H NMR spectroscopy was employed to study what effect exposing a poled device to air has upon the chemical structure of the helical metallopolymer, Figure S16. Poled films exposed to air were dissolved in CD_3CN and their ^{1}H NMR spectrum was recorded on a 500 MHz TCI cryoprobe spectrometer (to enhance the signal-to-noise for a small amount of metallopolymer in the thin film). A series of broad peaks were observed in the ^{1}H NMR spectrum (Figure S16c) as well as a collection of sharper signals that were not present in the pristine metallopolymer (Figure S16b). The chemical shifts of these signals do not correlate with those observed for the monomer unit, implying a different discrete species has been formed that contains protons in an aromatic environment as well as a formyl proton at 10.23 ppm. The difference in chemical shift may be due, in part, to the presence of paramagnetic Cu^{II} centres in the sample; however, the total number of defined signals observed is greater than expected for **A**. The presence of this formyl proton signal suggested that the organic strands have at least partially degraded after exposure to air and electric stress. We therefore hypothesise that these ^{1}H NMR signals correspond to degradation products of the helical strands in the presence of superoxide; a possible identity of the degradation product, based on the changes of the chemical shifts with respect to **A**, may be the formation of nitroso derivatives of **A**.[8] The generation of radical species may further account for the loss of ESR signal of the Cu^{II} centre. These radicals may coordinate with the Cu^{II} centres, satisfying the greater coordination number of Cu^{II}, and couple antiferromagnetically to the Cu^{II} states, resulting in an overall diamagnetic system, hence loss of the ESR signal.[9–11]

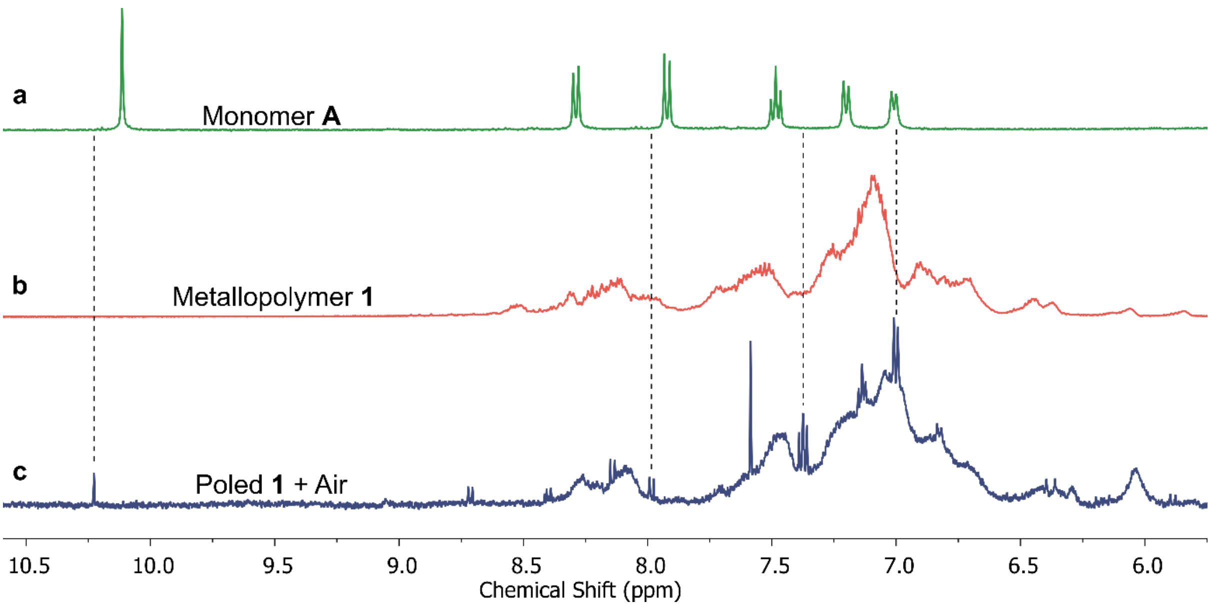

Figure S16. a, The ^{1}H NMR spectrum (500 MHz, 298 K, CD_3CN) of monomer **A**. **b**, The ^{1}H NMR spectrum (500 MHz, 298 K, CD_3CN) of metallopolymer **1**. Note the absence of signals corresponding to **A** in this spectrum. **c**, The ^{1}H NMR spectrum (500 MHz, 298 K, CD_3CN) of the material recovered from a poled device (containing **1**) that was exposed to air (this experiment was performed on a TCI cryoprobe spectrometer). Note the presence of defined signals in this spectrum; these signals do not correspond to the monomer unit. Dry, degassed CD_3CN was employed for all measurements.

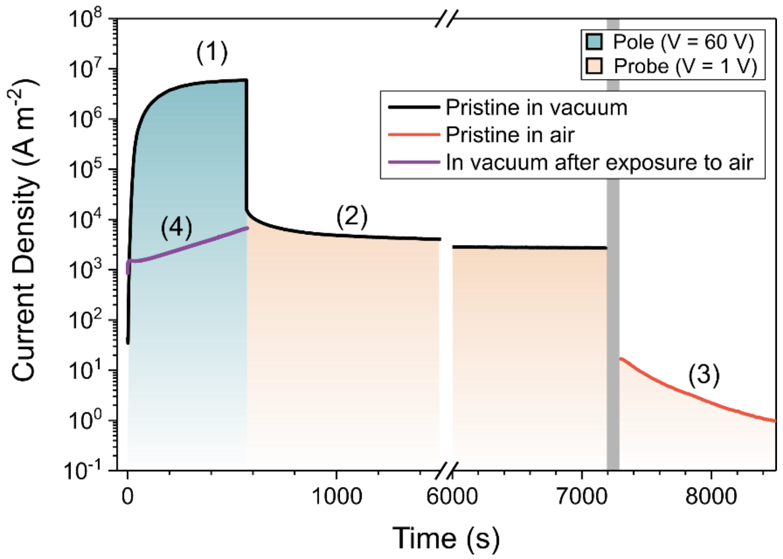

Figure S17. Current density as a function of time and voltage bias recorded on devices with structure as in Figure 1c (main manuscript), comprising a 50mer, enantiomerically enriched metallopolymer **1**. The black line shows the evolution in time of the current density upon application of subsequent pole (60 V) and probe (1 V) voltage steps, in vacuum (10^{-5} Bar). The same device was then exposed to air while held at 1V (red curve above 7000 s). The sample chamber was then evacuated and the current density measured again at 60 V on the same device (blue line). All the measurements were performed at 300 K. The numbers serve as a guide to the sequence in which the traces should be interpreted.

3.11 Electrochemistry of metallopolymer **1**

Longer helices require a greater oxidation potential than shorter oligomers to trigger the oxidation of Cu^{I} to Cu^{II}. Figure S18 below shows that the HOMO level plateaus after *ca.* 15 repeat units. We infer this length dependence of the HOMO level to result from the ease by which the structure of the metallopolymer can reorganise to support a Cu^{II} centre, i.e., the ease of axial compression of the double helix.

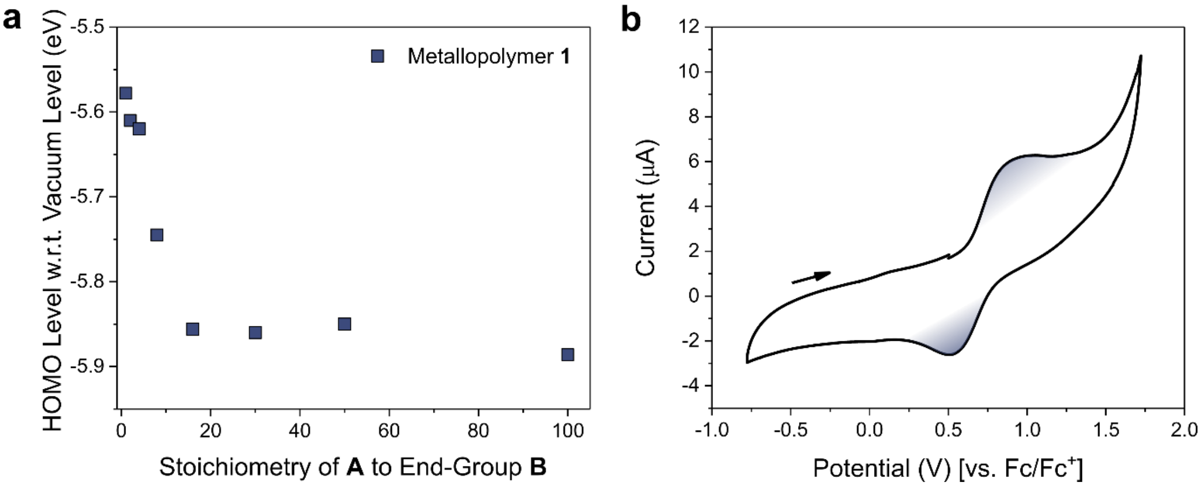

Figure S18. a, The HOMO levels (in eV) of oligomers of varying lengths, ranging from a 1mer to 100mer, of metallopolymer **1** were determined using cyclic voltammetry.[12] In each experiment, a ferrocene/ferrocenium redox couple (Fc/Fc^{+}) was measured as an internal standard and the resulting onsets of oxidation of the metallopolymers were referenced to this Fc/Fc^{+} couple. A 0.1 M $TBANTf_2$/MeCN electrolyte and a 100 mV s^{-1} scan rate was employed. The HOMO was determined using a potential value of 4.6 ± 0.2 eV for NHE versus vacuum level[13] and 0.63 eV for Fc/Fc^{+} versus NHE.[14] **b,** A representative CV plot of a 50mer of polymer **1** referenced against the Fc/Fc^{+} redox couple and recorded at 100 mV s^{-1}; the oxidation process was irreversible (the peak to peak separation, ΔE_p for a reversible process is 57 mV; ΔE_p for a irreversible process is >57 mV. Here we measure $\Delta E_p = 370$ mV). The irreversible nature of this electrochemical process implies a significant reorganisation of the chemical structure upon oxidation. The arrow indicates the scan direction; the oxidation and reduction peaks are shaded in the plot to guide the eye.

3.12 DFT and TD-DFT calculations

DFT calculations were performed on model compounds **4** and **5** to elucidate differences in their electronic properties and determine the nature of the HOMO level (Figure S19). These model compounds exhibit the key features of the helical metallopolymer: two conjugated helical strands comprised of imine linkages and ≥ 2 metal centres. Similar small compounds have been shown to be well described using B3LYP functionals with the triple-zeta basis set def2-tzvp and the RIJCOSX approximation with a def2-SVP/J auxiliary basis set.[15] Starting geometries were created by modifying a previously reported crystal structure[4] of a 3mer of **1**. Orca software was used to perform all electronic structure calculations.

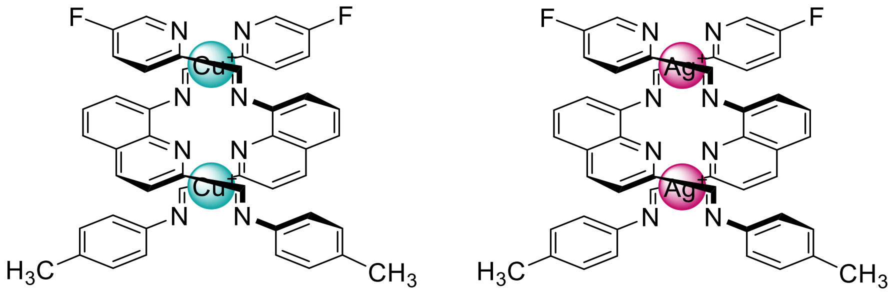

4 **5**

Figure S19. The chemical structures of model compounds **4** and **5** used in computational studies.

Time-Dependent DFT (TD-DFT) calculations were conducted using the ground state geometry-optimised configurations for **4** and **5** as obtained by DFT. The prediction of the lowest-energy transitions correlates well to the observed bands in UV-vis spectra of **4** and **5**. Theory correctly predicts the absence of an MLCT in the case of **5**, whereas **4** displays a low energy MLCT state, showing that the HOMO is localised on the Cu-core. Both transitions involve the transfer of an electron to the helical ligand, displayed in Table S3 below. This prediction of a photoexcited radical anion present on the ligand strand is supported by transient absorption measurements (Figure S10).

Table S3. Results of TD-DFT calculations performed on helical model compounds. A representation of the change in electron density for the lowest energy transition is displayed (green represents a gain in electron density, purple represents a loss of electron density), in addition to the predicted lowest energy transition.

	Electron Difference Density Plot	Lowest Excitation Energy (nm)
1mer **4**	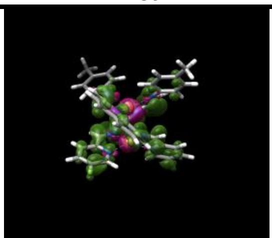	728.4
1mer **5**	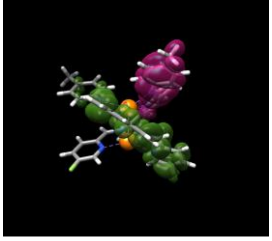	511.5

3.13 Spectroelectrochemistry of metallopolymer **1**

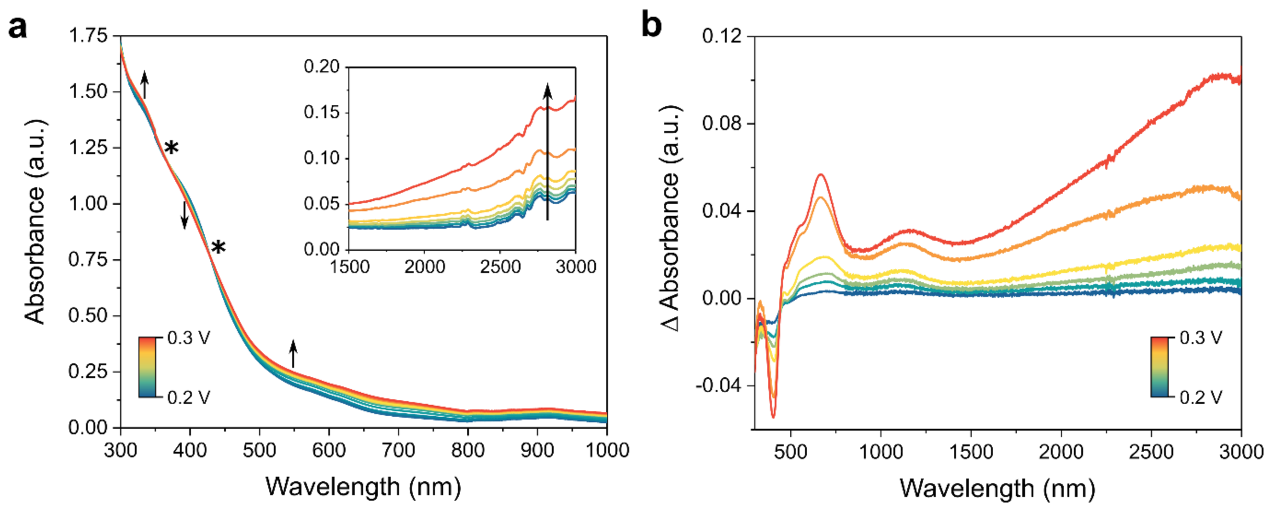

Figure S20. a, The UV-vis spectrum as the external potential applied to **1** is increased, as obtained by spectroelectrochemistry performed in a 0.5 M TBANTf$_2$/MeCN (TBA = *n*Bu$_4$N$^+$) electrolyte at 298 K under N$_2$. Isosbestic points are highlighted by asterisks, while the directions of change in band intensities are shown with arrows. The inset shows the appearance of a new band in the NIR region of the spectrum upon oxidation. These changes are consistent with Cu^{II} formation and inter-valence charge transfer (IVCT) between Cu^{I}-Cu^{II} sites. **b**, The change in absorbance as a function of oxidation potential, showing a clear increase in the IVCT band at *ca*. 3000 nm. No degradation of the electrolyte was observed between 0 V to 0.4 V.

3.14 Control measurements for spectroelectrochemical study

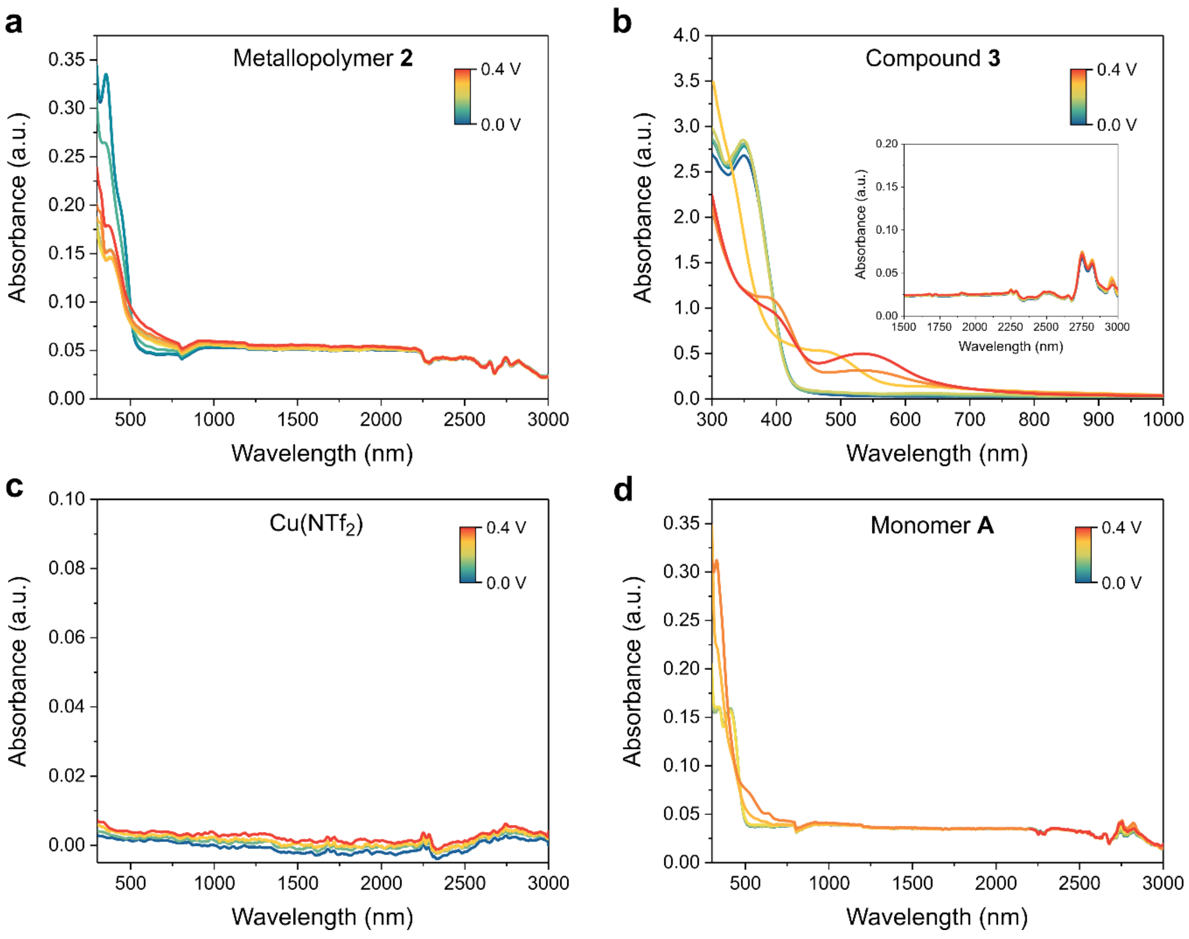

Figure S21. Control spectroelectrochemical measurements were carried out to support the assignment of changes observed in Figure S20. Measurements were carried out at 298 K in an optically transparent thin layer electrochemical (OTTLE) cell using a 0.5 M $TBANTf_2$ electrolyte in dry, degassed MeCN. No degradation of the electrolyte was observed over the potential range employed. The potentials shown in the plot should be interpreted qualitatively as they are referenced against an Ag/AgCl pseudo-reference electrode. **a**, Polymer **2** did not show the onset of a low energy absorption band upon oxidation, instead only changes to the ligand centred bands were observed. **b**, Model compound **3** did not show any appearance of a low energy intervalence charge-transfer band upon oxidation.[15] **c**, The UV-vis plot of the $CuNTf_2$ salt showed no changes upon oxidation. **d**, Upon oxidation, monomer **A** exhibited changes in the absorption bands associated with π-π* star transitions in the organic ligand. Further oxidation beyond 0.4 V resulted in the precipitation of material (for a, b, d).

4 Calculations

4.1 Calculation of conductivities

Conductivity, σ (S m^{-1}), can be calculated from the following equation,

$$\sigma = \frac{J}{E}$$

where *J* is the magnitude of the current density (A m^{-2}), *E* is the magnitude of the electric field (V m^{-1}). The distance between the electrodes in the device shown in Figure 1c (main manuscript) is 10 µm. The device in Figure 1e in the main manuscript reached a current density of 2.0×10^{7} A m^{-2} at 100 V, corresponding to a conductivity of 2.0 S m^{-1}. The devices in Figure 2a-c at 60 V reached current densities of 1.0×10^{7} A m^{-2}, 1.2×10^{7} A m^{-2} and 1.4×10^{7} A m^{-2} respectively, corresponding to conductivities of 1.7, 2.0 and 2.3 S m^{-1} respectively.

4.2 Calculation of number of spins (ESR measurements)

The average volume occupied by a copper atom of the metallopolymer was estimated by considering space-filling rectangular cuboids, and using the crystal structure[4] of a 3mer of **1** (containing four Cu^{I} centres).[4] The volume, *V*, of a cuboid containing a copper atom is,

$$V = H \times L^2$$

where the height, *H*, is the separation between Cu centres 0.28 nm and the side L is 1.21 nm, giving a volume of 4.1×10^{-28} m^{-3} per copper atom The active volume of the device (i.e. excluding the areas not in between the electrodes) was calculated based on the following dimensions: a width of 1.8 mm, a total length of 13.3 mm and a height of 50 nm. This affords an active volume of 1.2×10^{-12} m^{-3}. The number of copper atoms in this volume is therefore 2.9×10^{15}. Given the total number of spins measured in the poled device (8×10^{14}), the number of copper atoms per spin is therefore 3.7: this corresponds to 27% of copper atoms being in the Cu^{II} state.

5 References

[1] G. Jayamurugan, D. A. Roberts, T. K. Ronson, J. R. Nitschke, *Angew. Chemie - Int. Ed.* **2015**, *54*, 7539.

[2] M. Wolffs, S. J. George, Ž. Tomović, S. C. J. Meskers, A. P. H. J. Schenning, E. W. Meijer, *Angew. Chemie - Int. Ed.* **2007**, *46*, 8203.

[3] Zetasizer software version 7.03, *Zetasizer Softw. version 7.03, Malvern Instruments, Malvern, UK* **n.d.**

[4] J. L. Greenfield, F. J. Rizzuto, I. Goldberga, J. R. Nitschke, *Angew. Chemie - Int. Ed.* **2017**, *56*, 7541.

[5] J. L. Greenfield, E. W. Evans, D. Di Nuzzo, M. Di Antonio, R. H. Friend, J. R. Nitschke, *J. Am. Chem. Soc.* **2018**, *140*, 10344.

[6] J. M. Holland, X. Liu, J. P. Zhao, F. E. Mabbs, C. A. Kilncr, M. Thornton-Petf, M. A. Halcrow, *J. Chem. Soc. Dalt. Trans.* **2000**, 3316.

[7] G. R. Eaton, S. S. Eaton, D. P. Barr, R. T. Weber, *Quantitative EPR: A Practitioners Guide*, Springer Vienna, Vienna, **2010**.

[8] E. Pretsch, P. Bühlmann, M. Badertscher, *Structure Determination of Organic Compounds: Tables of Spectral Data*, **2009**.

[9] A. Godiksen, F. N. Stappen, P. N. R. Vennestrøm, F. Giordanino, S. B. Rasmussen, L. F. Lundegaard, S. Mossin, *J. Phys. Chem. C* **2014**, *118*, 23126.

[10] S. S. Eaton, K. M. More, B. M. Sawant, G. R. Eaton, *J. Am. Chem. Soc.* **1983**, *105*, 6560.

[11] F. Thomas, O. Jarjayes, C. Duboc, C. Philouze, E. Saint-Aman, J. L. Pierre, *Dalt. Trans.* **2004**, 2662.

[12] D. Veldman, S. C. J. Meskers, R. A. J. Janssen, *Adv. Funct. Mater.* **2009**, *19*, 1939.

[13] J. O. Bockris, S. U. M. Khan, *Surface Electrochemistry*, **1993**.

[14] V. V. Pavlishchuk, A. W. Addison, *Inorganica Chim. Acta* **2000**, *298*, 97.

[15] B. Dicke, A. Hoffmann, J. Stanek, M. S. Rampp, B. Grimm-Lebsanft, F. Biebl, D. Rukser, B. Maerz, D. Göries, M. Naumova, M. Biednov, G. Neuber, A. Wetzel, S. M. Hofmann, P. Roedig, A. Meents, J. Bielecki, J. Andreasson, K. R. Beyerlein, H. N. Chapman, C. Bressler, W. Zinth, M. Rübhausen, S. Herres-Pawlis, *Nat. Chem.* **2018**, *10*, 355.
